# Supplementary material for: Spatially resolved mapping of proteome turnover dynamics with subcellular precision
Source: Nat Commun. 2023 Nov 8;14:7217. doi: 10.1038/s41467-023-42861-8 (PMC10632371; doi:10.1038/s41467-023-42861-8)
Supplement: Supplementary file 1 — Supplementary Information [file 41467_2023_42861_MOESM1_ESM.pdf]

Supplementary Figures

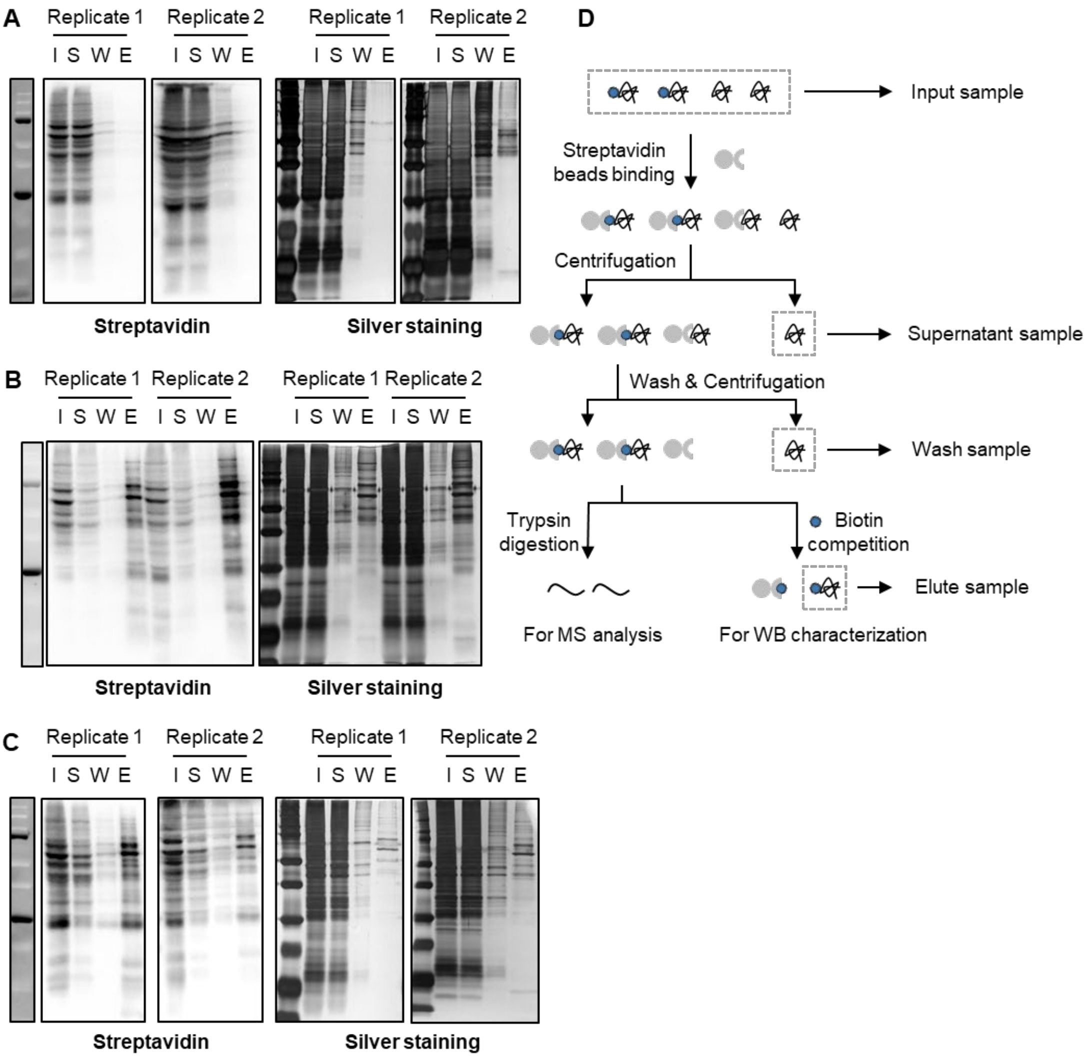

Supplementary Figure 1. Immunoblotting and silver staining characterization of Prox-SILAC labeling in HEK293T mito-APEX2 cell line. (A-C) Western blots (left) and silver staining (right) of enriched proteins in HEK293T cells stably expressing Mito-APEX2 with 4 hr prox-SILAC (**A**), 8 hr prox-SILAC (**B**), 12 hr prox-SILAC (**C**) I: input; S: supernatant; W: wash; E; elution. **(D)** Scheme for western blot sample preparation. Source data are provided as a Source Data file

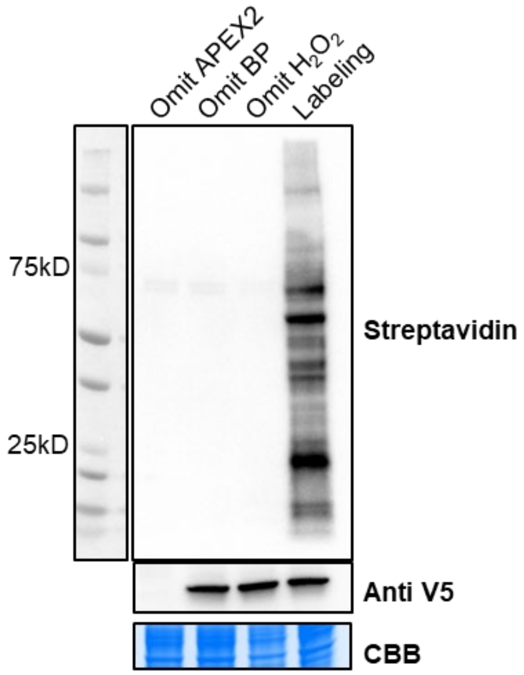

Supplementary Figure 2. Streptavidin-HRP blot analysis of protein biotinylation in HEK293T cells expressing mito-APEX2. Source data are provided as a Source Data file

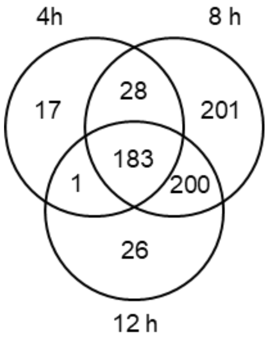

Supplementary Figure 3. Venn diagram showing the overlap of quantified proteins in 4 hr, 8 hr and 12 hr prox-SILAC labeling in mito-APEX2 cells.

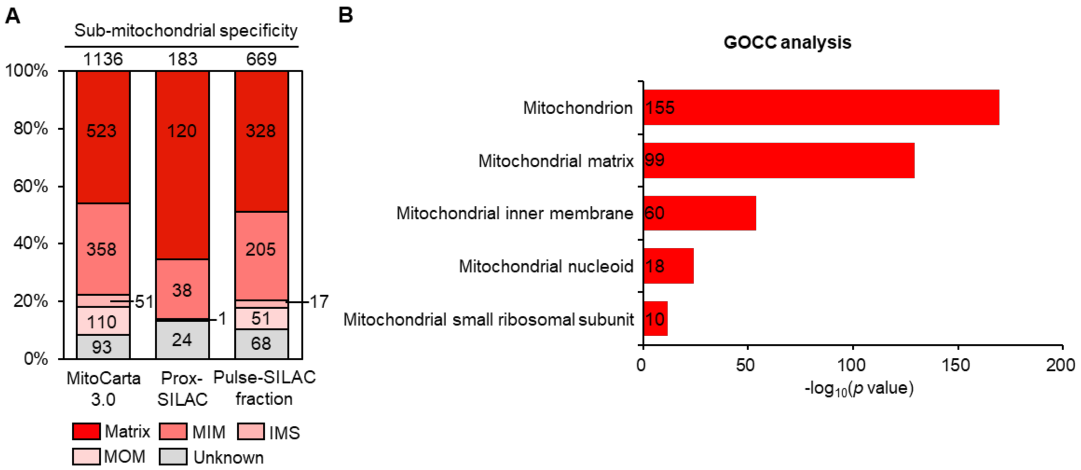

Supplementary Figure 4. Mitochondrial specificity analysis of mito-APEX2 prox-SILAC dataset. (A) Sub-mitochondrial specificity analysis of our mito-prox-SILAC dataset, comparing with Mitocarta 3.0 database and previous work (pulse-SILAC with mitochondria isolation) [1]. **(B)** GOCC analysis of the 162 proteins identified in our mitochondrial dataset with previous mitochondrial annotations. The numbers of proteins with corresponding GOCC annotations are list on the left of the chart.

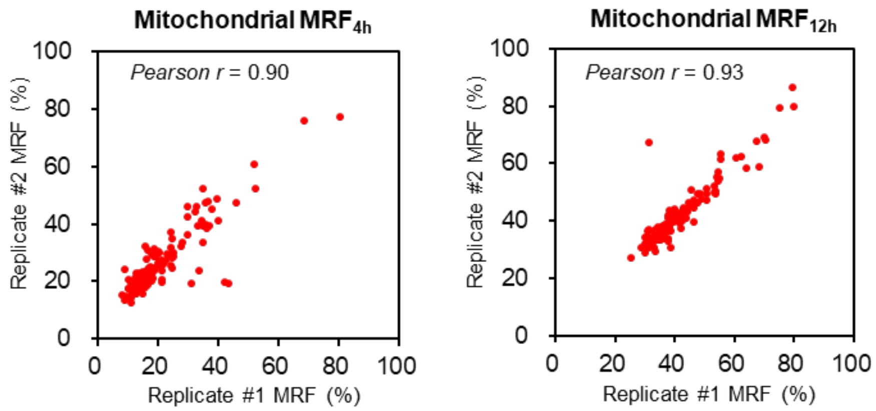

Supplementary Figure 5. Scatter plots showing the MRF values (left: 4hr; right: 12hr) of mitochondrial proteins identified from replicated experiments.

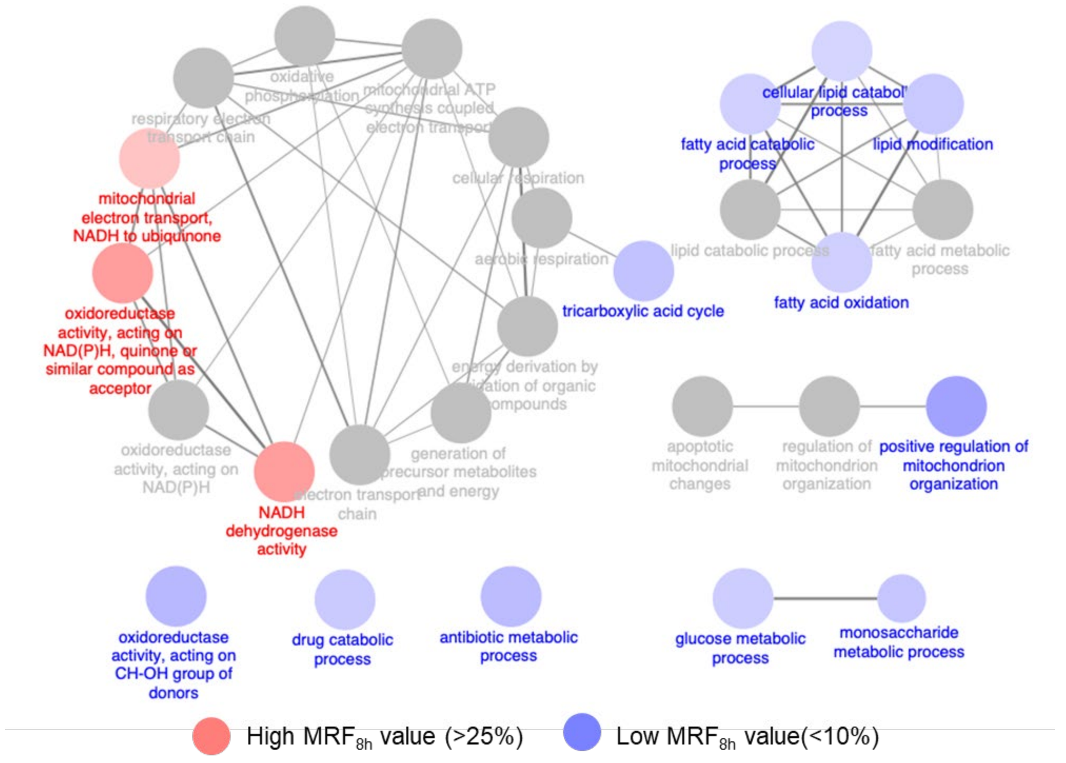

Supplementary Figure 6. GOBP clusters of identified mitochondrial proteins. The red and blue clusters indicate biological processes involving faster and slower turnover proteins, respectively.

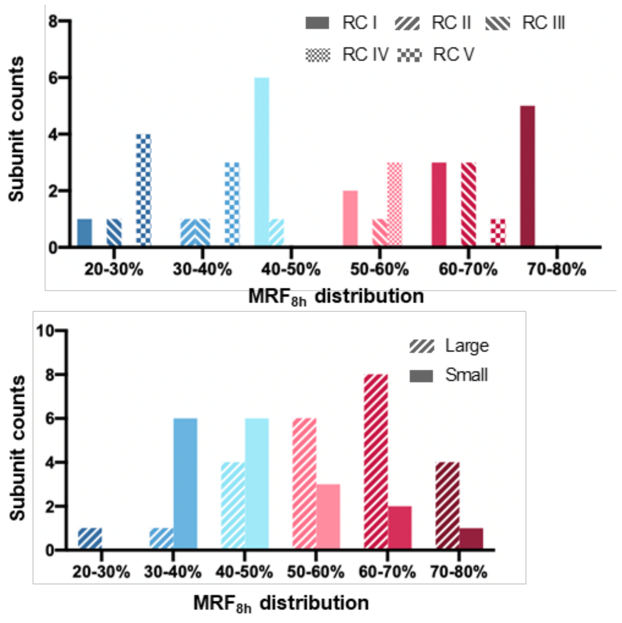

Supplementary Figure 7. MRF_{8h} distribution of respiratory complexes (RC I-V, top) and mitochondrial ribosome large and small subunits (bottom).

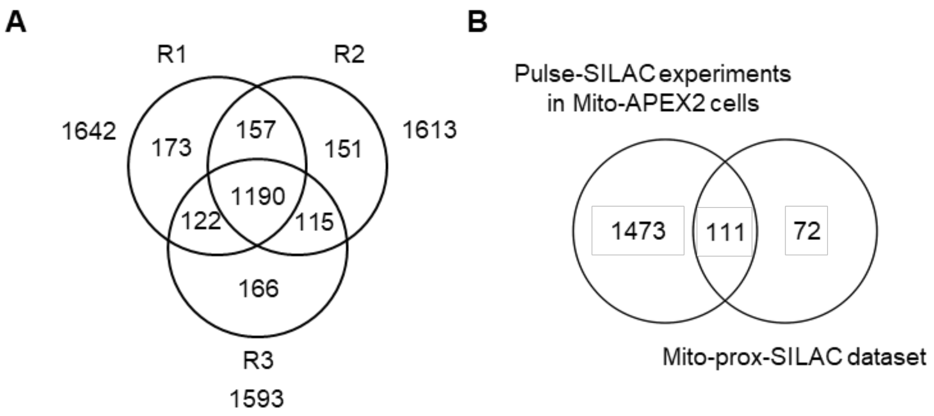

Supplementary Figure 8. Venn diagrams showing the overlap of quantified proteins in replicated pulse-SILAC experiments in HEK293T cells. (A) Venn diagram showing the overlap of three replicates. **(B)** Overlap of 111 proteins identified both in pulse-SILAC and prox-SILAC.

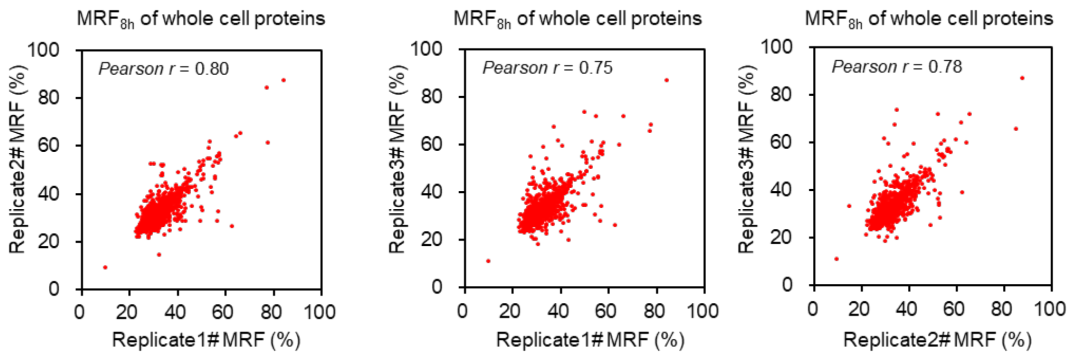

Supplementary Figure 9. Scatter plots showing the MRF_{8h} values measured in replicated pulse-SILAC experiments in HEK293T cells.

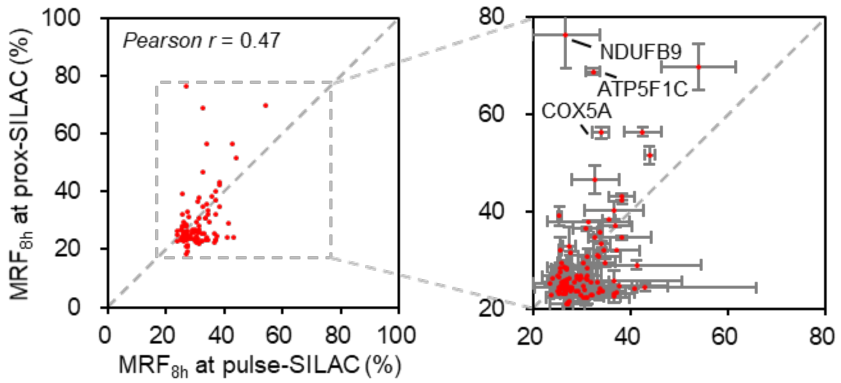

Supplementary Figure 10. Scatter plot of MRF_{8h} values of mitochondrial proteins measured in mito-APEX2 prox-SILAC versus pulse-SILAC. The zoom-in view with error bars is shown on the right.

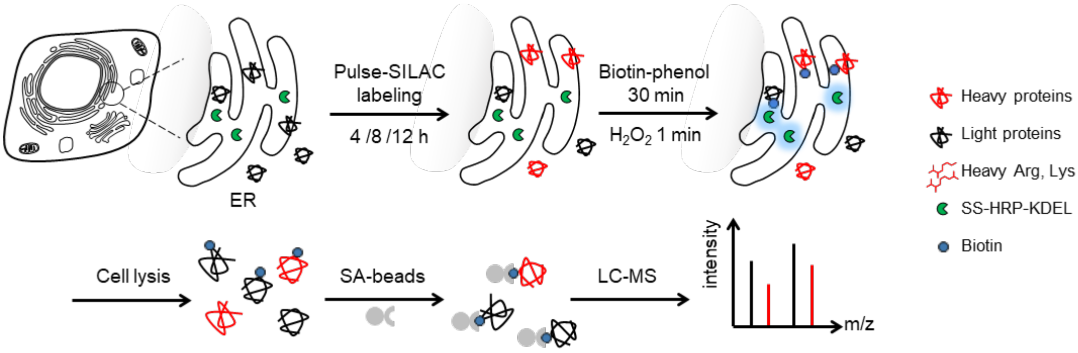

Supplementary Figure 11. Experimental scheme of prox-SILAC in HEK293T SS-HRP-KDEL cells. HRP was targeted to ER lumen in HEK293T cells and stable cells were initially cultured in light medium. To tag the nascent proteins, cells were briefly cultured in heavy SILAC medium for various durations. To label the ER proteome, cells were incubated with biotin-phenol and hydrogen peroxide to initiate the proximity labeling reaction. The nascent ER proteins were thus doubly labeled with both biotin and heavy arginine/lysine. Following cell lysis, biotinylated ER proteins were enriched by streptavidin-coated beads, trypsin-digested and analyzed by LC-MS/MS.

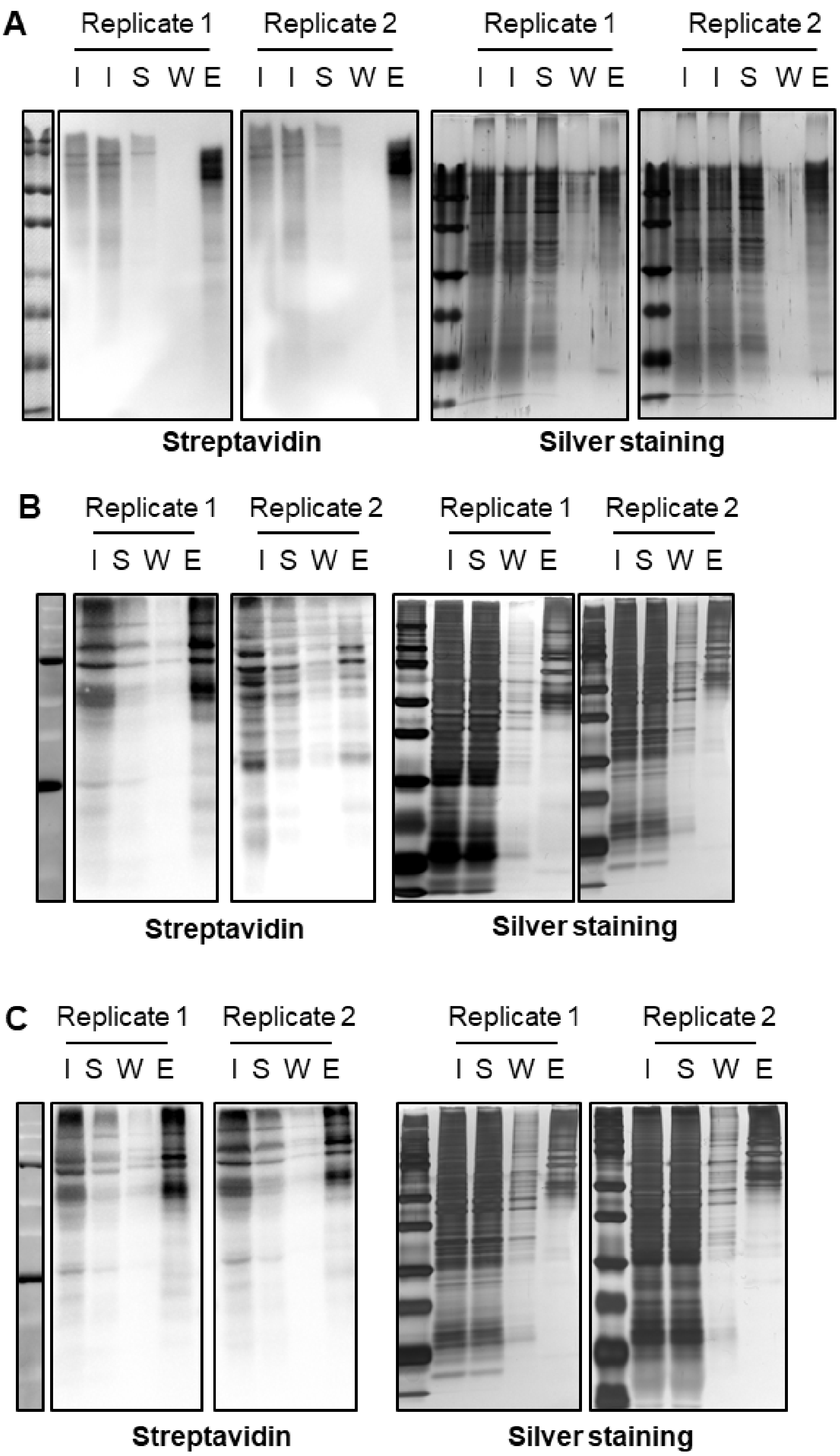

Supplementary Figure 12. Immunoblotting and silver staining characterization of ER prox-SILAC labeling in HEK293T cells. Western blots (left) and silver staining (right) of enriched proteins at 4 hr (**A**), 8 hr (**B**) and 12 hr (**C**) prox-SILAC labeling. I: input; S: supernatant; W: wash; E; elution. Source data are provided as a Source Data file

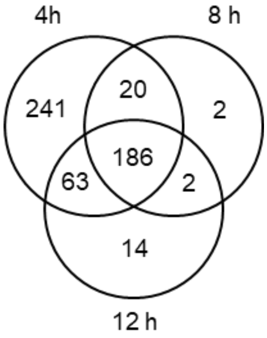

Supplementary Figure 13. Venn diagram showing the overlap of quantified proteins in 4 hr, 8 hr and 12 hr ER prox-SILAC labeling in HEK293T cells.

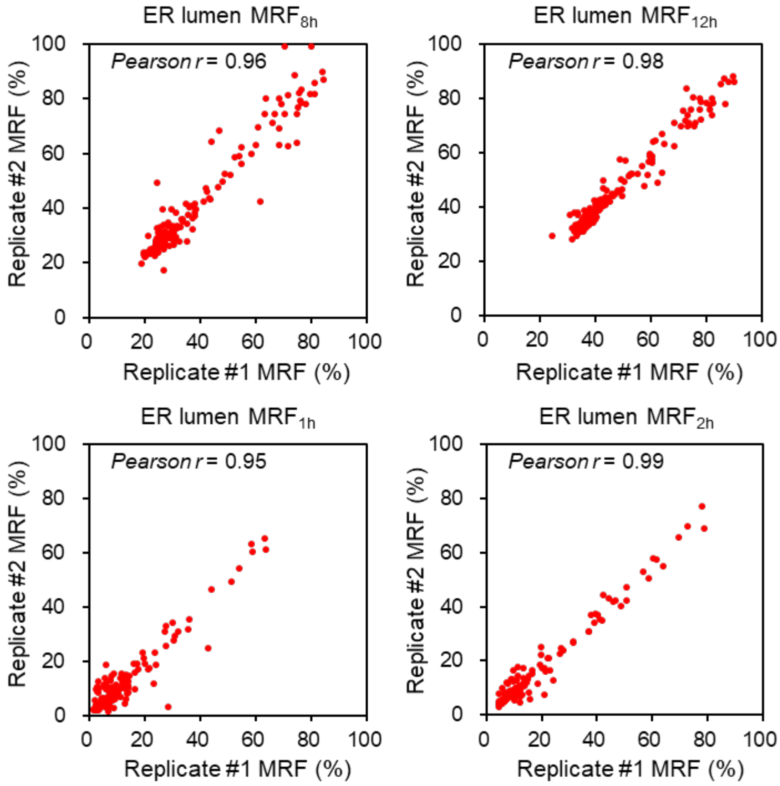

Supplementary Figure 14. Scatter plots showing the MRF values of ER proteins identified from replicated experiments. Top row: MRF_{8h} (left) and MRF_{12h} (right) values of proteins identified from the overlap of replicated experiments at three time points (4 hr, 8hr, 12hr). Bottom row: MRF_{1h} (left) and MRF_{2h} (right) values of proteins identified from replicated experiments at five time points (1 hr, 2 hr, 4 hr, 8hr, 12hr).

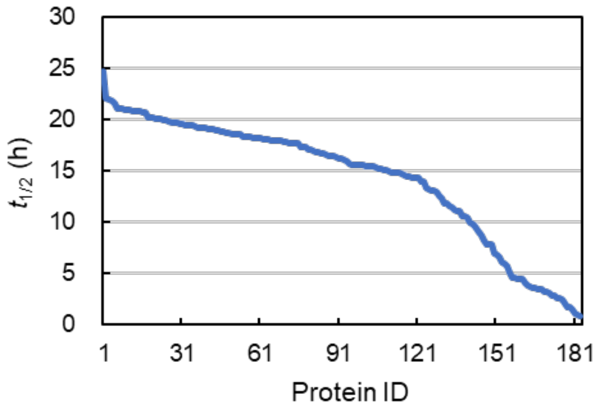

Supplementary Figure 15. The distribution of half-lives of 183 ER proteins identified in 1 hr, 2 hr, 4 hr, 8hr and 12hr ER prox-SILAC experiments.

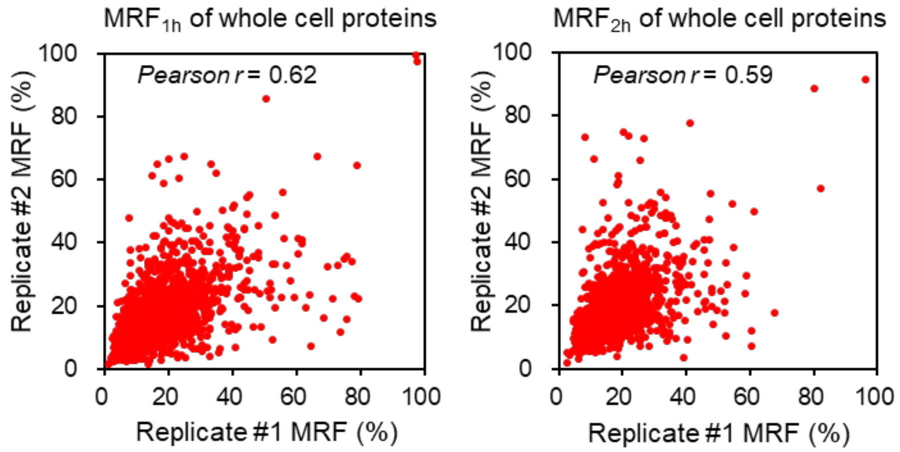

Supplementary Figure 16. Scatter plots showing the MRF values of proteins identified from replicated pulse-SILAC experiments at 1 hr (MRF_{1h}, left) and 2 hr (MRF_{2h}, right).

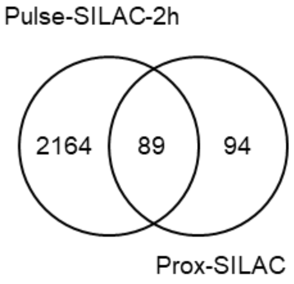

Supplementary Figure 17. Venn diagram showing the overlap of quantified proteins in 2 hr pulse-SILAC experiments and our ER prox-SILAC dataset.

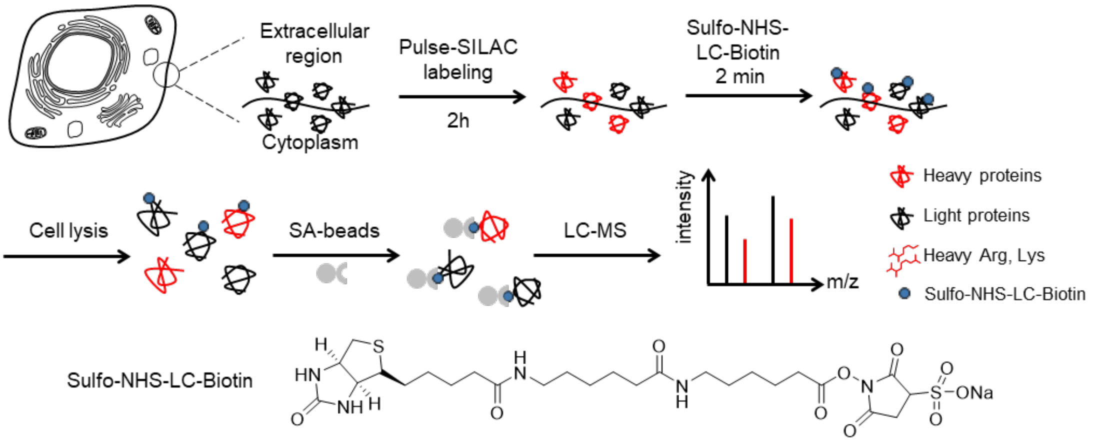

Supplementary Figure 18. Experimental scheme of cell surface pulse-SILAC in HEK293T cells. To tag the nascent proteins, HEK293T cells were briefly cultured in heavy SILAC medium for 2 hr. Cells were incubated with sulfo-NHS-LC-biotin for 10 mins to capture the cell surface proteins. The nascent PM proteins were thus doubly labeled with both biotin and heavy arginine/lysine. Following cell lysis, biotinylated proteins were enriched by streptavidin-coated beads, trypsin-digested and analyzed by LC-MS/MS.

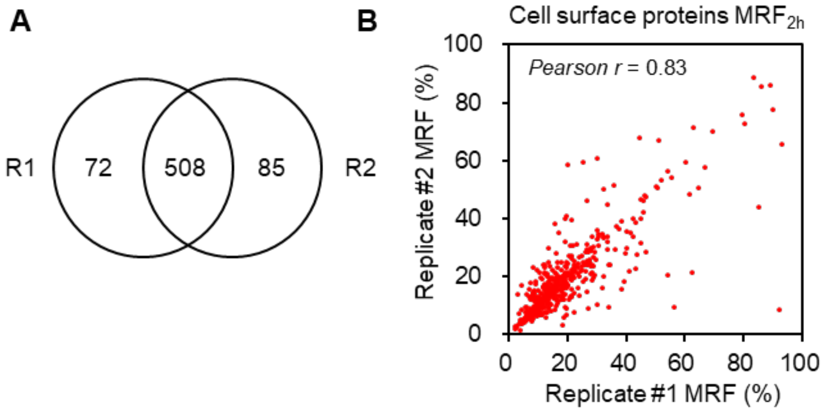

Supplementary Figure 19. Pulse-SILAC experiments on the cell surface. (A) Venn diagram showing the overlap of proteins identified in duplicated cell surface pulse-SILAC experiments. **(B)** Scatter plot showing the MRF values of the two replicates.

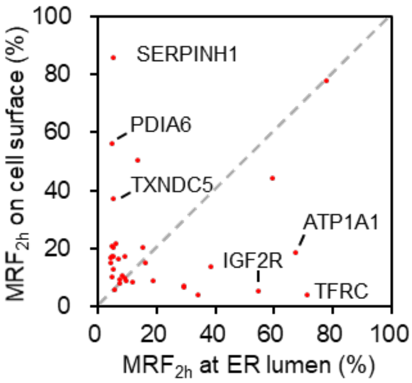

Supplementary Figure 20. Scatter plot of MRF_{2h} values of secretory pathway proteins measured in ER prox-SILAC against cell surface pulse-SILAC.

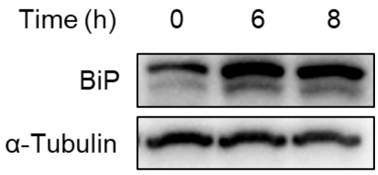

Supplementary Figure 21. Immunoblotting analysis of thapsigargin-induced ER stress. HeLa-SS-HRP-KDEL cells were treated with 1 μM thapsigargin for 6 – 8 hr. Top: anti-BiP, which is a marker for ER stress. Bottom: anti-tubulin, which is a loading marker. Source data are provided as a Source Data file

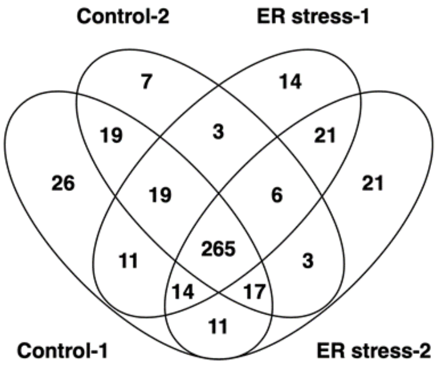

Supplementary Figure 22. Venn diagram showing the overlap of quantified proteins in replicated ER prox-SILAC experiments in HeLa cells, in the presence and absence of ER stress.

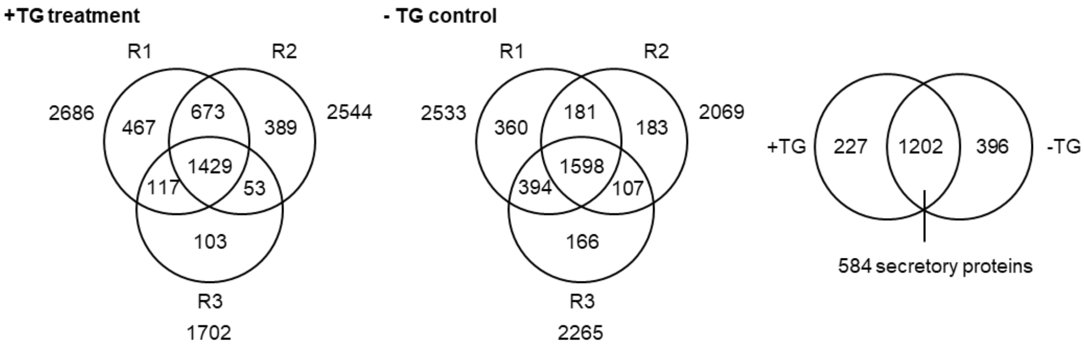

Supplementary Figure 23. Venn diagrams showing the overlap of quantified proteins in replicated 2 hr pulse-SILAC experiments in HeLa cells with or without thapsigargin (TG) treatment. Left: the overlap of the three replicates with TG treatment; middle: the overlap of the three replicates without TG treatment; right: the overlap of +TG triplicates and -TG triplicates.

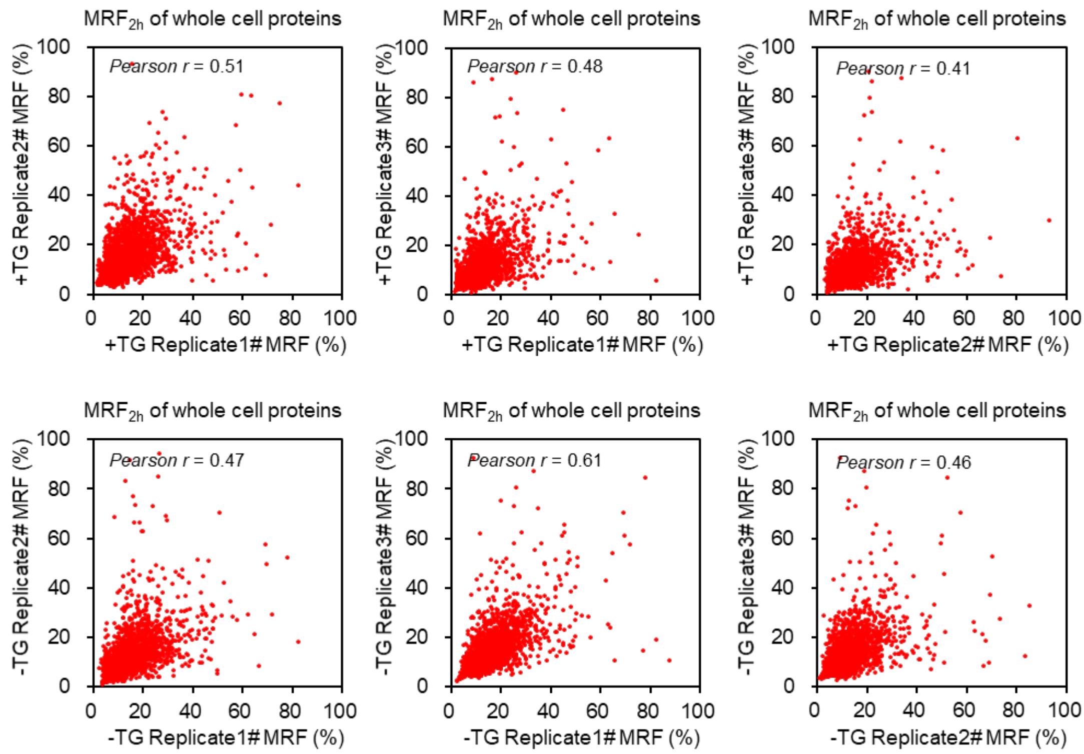

Supplementary Figure 24. Scatter plots showing the MRF values of proteins identified in replicated pulse-SILAC experiments in HeLa cells. Top and bottom rows show MRF_{2h} values of proteins identified from replicated +TG and -TG experiments, respectively.

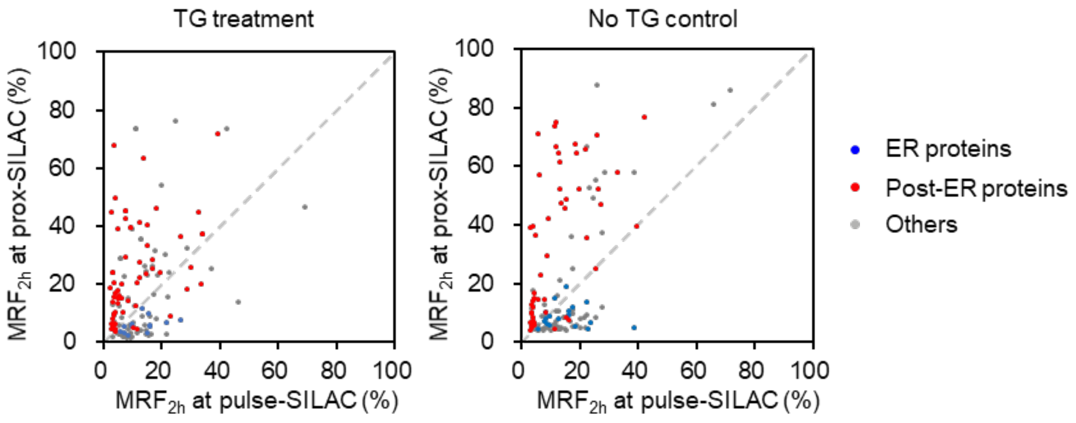

Supplementary Figure 25. Scatter plot of MRF_{2h} values of ER proteins measured in ER prox-SILAC against whole-cell pulse-SILAC in HeLa-SS-HRP-KDEL cells in the presence and absence of thapsigargin (TG) treatment. Red dots represent post-ER trafficking proteins (i.e. cell membrane, Golgi apparatus, and lysosome). Blue dots represent ER resident proteins.

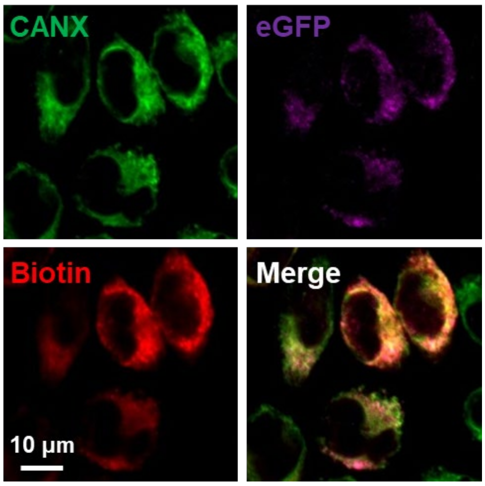

Supplementary Figure 26. Immunofluorescence characterization of the localization of HRP-eGFP-KDEL in SH-SY5Y cells. The expression of ER marker calnexin, HRP-eGFP-KDEL, and biotinylated proteins are shown in green, magenta, and red, respectively. Scale bar, 10 μm. The image is a were representative field from at least three fields in one experiment.

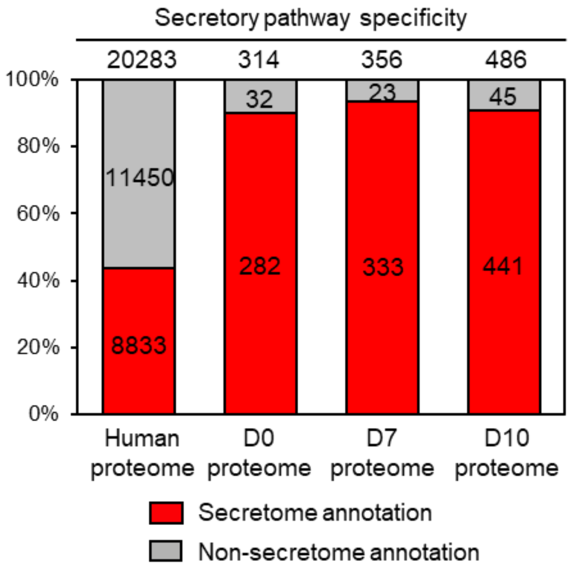

Supplementary Figure 27. Secretory pathway specificity analysis for ER prox-SILAC proteomic data at D0, D7 and D10. Red bars and numbers indicate proteins with prior secretory annotations in the GOCC database. Left: entire human proteome (from Uniprot database). The three columns on the right show proteins in D0, D7 and D10 datasets.

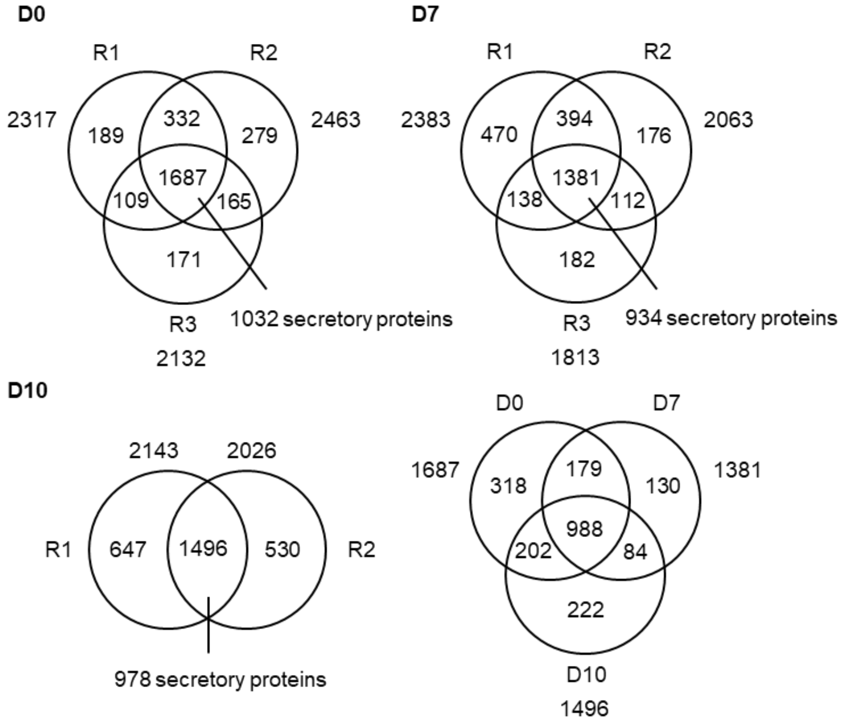

Supplementary Figure 28. Venn diagrams showing the overlap of quantified proteins in replicated 2 hr pulse-SILAC experiments in SH-SY5Y-SS-HRP-KDEL cells at various stages of differentiation. Bottom right: the overlap of proteins identified at D0, D7 and D10.

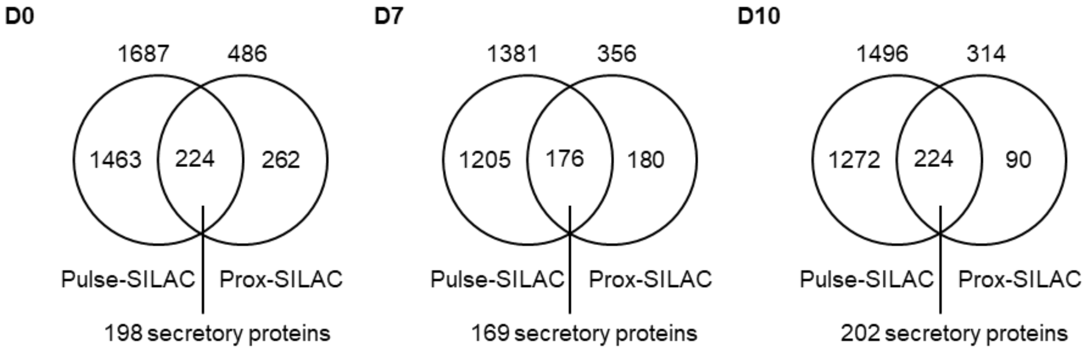

Supplementary Figure 29. Venn diagrams showing the overlap of quantified proteins in 2 hr pulse-SILAC experiments and ER prox-SILAC experiments in SH-SY5Y-SS-HRP-KDEL cells during differentiation.

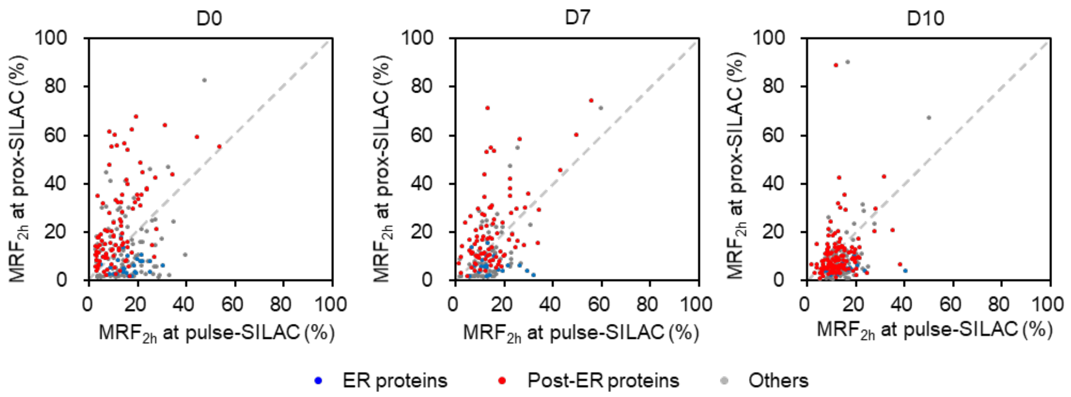

Supplementary Figure 30. Scatter plot of MRF_{2h} values of ER proteins measured in ER prox-SILAC against whole-cell pulse-SILAC in SH-SY5Y-SS-HRP-KDEL cells during differentiation. Red dots represent post-ER trafficking proteins (i.e. cell membrane, Golgi apparatus, and lysosome). Blue dots represent ER resident proteins.

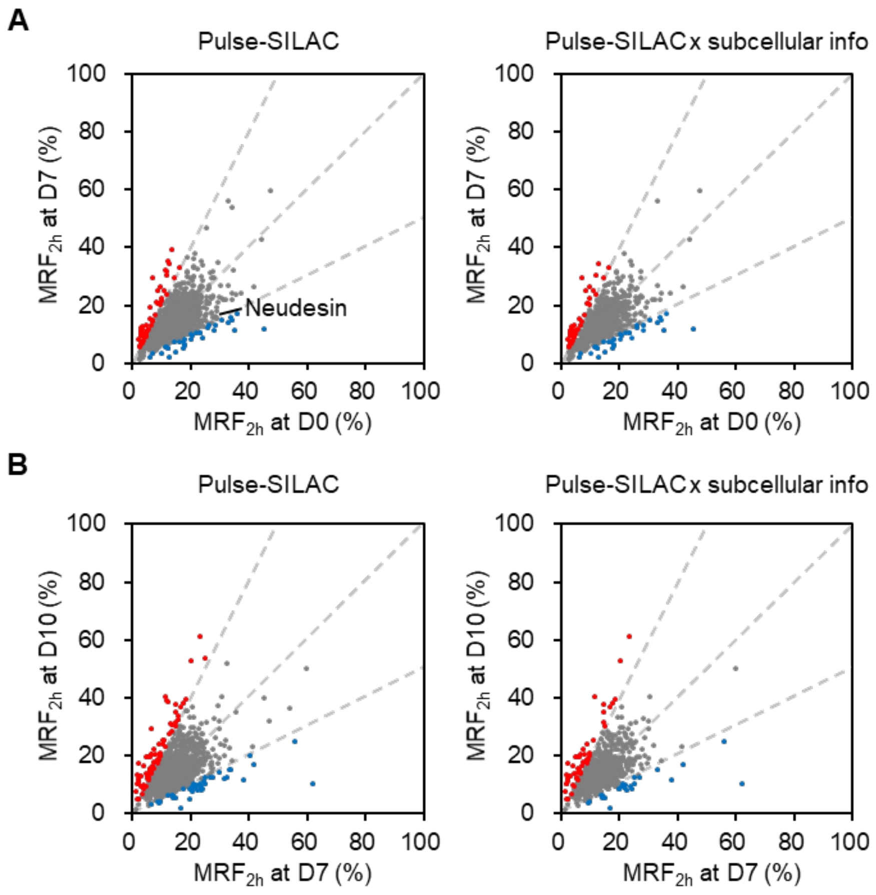

Supplementary Figure 31. Scatter plots comparing the MRF_{2h} values of proteins identified in pulse-SILAC experiments at different stages of SH-SY5Y differentiation. (A) Left: pulse-SILAC MRF_{2h} values at D0 and D7. Right: MRF_{2h} values of proteins with secretome annotations. **(B)** Left: pulse-SILAC MRF_{2h} values at D7 and D10. Right: MRF_{2h} values of proteins with secretome annotations. Red and blue dots represent proteins with 2-fold higher and lower MRF_{2h} values, respectively.

Supplementary Methods

Reagents, antibodies, and plasmids used in this study are summarized below:

REAGENT or RESOURCE	SOURCE	IDENTIFIER
**Antibodies**		
Mouse anti-TOMM20	Abcam	Cat# ab56783; RRID: AB_945896
Rabbit anti-V5	Abcam	Cat# ab9116; RRID: AB_307024
Rabbit anti-calnexin	Abcam	Cat# ab22595; RRID: AB_2069006
Rabbit anti-Bip	Abcam	Cat# ab32618; RRID: AB_732737
Chicken anti-MAP2	Abcam	Cat# ab5392; RRID: AB_2138153
Mouse anti-HA	Biodragon	Cat# B1003
Mouse anti-V5	Biodragon	Cat# B1005
Mouse anti-GFP	Biodragon	Cat# B1152
Goat anti-rabbit Alexa Flour 488	Invitrogen	Cat# A11034; RRID: AB_2576217
Goat anti-rabbit Alexa Flour 568	Invitrogen	Cat# A11036; RRID: AB_10563566
Goat anti-mouse Alexa Flour 488	Invitrogen	Cat# A11029; RRID: AB_2534088
Goat anti-mouse Alexa Flour 568	Invitrogen	Cat# A11031; RRID: AB_144696
Goat anti-chicken Alexa Flour 488	Invitrogen	Cat# A11039; RRID: AB_2534096
Streptavidin-Alexa Fluor 647	Invitrogen	Cat# S21374
Streptavidin-HRP conjugate	Invitrogen	Cat# 21124
HRP-conjugated goat anti mouse IgG	Biodragon	Cat# BF03001
**Chemicals, Peptides, and Recombinant Proteins**		
Phanta® Max Super-Fidelity DNA Polymerase	Vazyme	Cat# P505-d2
2×Pfu MasterMix (Dye)	Cwbio	Cat# CW0686A
DMEM medium	Gibco	Cat# C11995500BT
Fetal bovine serum	Gibco	Cat# 100099044
0.25% Trypsin-EDTA	Gibco	Cat# 25200056
Lipofectamine 3000 Reagent	Invitrogen	Cat# L3000008
Opti-MEM	Gibco	Cat# 31985062
Blasticidin	Selleck	Cat# S7419
D-Biotin	TCI	Cat# B0463
N-hydroxysuccinimide	J&K	Cat# 117997

1-(3-dimethylaminopropyl)-3-ethylcarbodiimide hydrochloride	J&K	Cat# 211112
N,N-Dimethylformamide	J&K	Cat# 966438
Ethanol	Tongguang	Cat# 104022
Tyramine	J&K	Cat# 953409
Triethylamine	J&K	Cat# 432915
Methanol	Tongguang	Cat# 104028
Matrigel	Corning	Cat# 354248
Biotin-phenol	This paper	N/A
Dimethyl sulfoxide	Sigma	Cat# D5879
Hydrogen peroxide	Tongguang	Cat# 106057
Sodium azide	Amresco	Cat# 0639-250G
Sodium ascorbate	Aladdin	Cat# S105024
Trolox	Sigma	Cat# 238813-5G
40% formaldehyde	Aladdin	Cat# F111934-500ml
PBS buffer	Solarbio	Cat# P1020-500ml
Tween-20	Solarbio	Cat# T8220
Triton X-100	Tongguang	Cat# 106094
DAPI	Invitrogen	Cat# D1306
L-arginine-$^{13}C_6$-$^{15}N_4$	Cambridge isotope laboratories	Cat# CNLM-539-H-PK
L-lysine-$^{13}C_6$-$^{15}N_2$	Cambridge isotope laboratories	Cat# CNLM-291-H-PK
SILAC-DMEM medium	Invitrogen	Cat# 88364
SILAC-FBS	Gibco	Cat# 26400044
Tris base	Sigma	V900483
Sodium chloride	Tongguang	Cat# 112008
NP-40	Aladdin	Cat# N274337-500ml
Sodium deoxycholate	Sigma	Cat# 30970-25G
Sodium dodecyl sulfate	Solarbio	Cat# S8010
Protease inhibitors cocktail	Roche	Cat# 04693132001
PVDF membrane	Bio-Rad	Cat# 1620177
Clarity western ECL substrate	Bio-Rad	Cat# 1705060
Bovine albumin	Sangong	Cat# A600332-0100
Streptavidin agarose resin	Pierce	Cat# 20228
Urea	Sigma	Cat# U1250-1KG
Dithiothreitol	Sigma	Cat# D9163
Iodoacetamide	Sigma	Cat# I6125
Triethylammonium bicarbonate buffer	Sigma	Cat# T7408
Sequencing-grade trypsin	Promega	Cat# V5111
Acetonitrile	Fisher	Cat# A998-4
Trifluoroacetic acid	Macklin	Cat# T818782-100ml
1M Tris-HCl buffer, pH ~ 7.5	Invitrogen	Cat# 15567-027

Calcium chloride	Xilong	Cat# S1669
Trypsin	Sigma	Cat# T1426
Formic acid	Fluka	Cat# 94318
Thapsigargin	J&K	Cat# 552112
All-trans-retinoic acid	MCE	Cat# HY-14649
Brain-derived neurotrophic factor	Peprotech	Cat# 450-02-10
**Critical Commercial Assays**		
Lightening clone kit	Biodragon	Cat# BDIT0014
Gel extraction kit	Omega	Cat# D2500-02
EndoFree Mini Plasmid Kit II	Tiangen	Cat# DP118-02
BCA Protein Assay Kit	Pierce	Cat# 23227
High pH reversed-phase peptide fractionation kit	Pierce	Cat# 84868
**Experimental Models: Cell Lines**		
WT-human embryonic kidney 293T cell line	American type culture collection, ATCC	N/A
WT-HeLa cell line	Chinese biomedical experimental cell bank	N/A
WT-SH-SY5Y cell line	Chinese biomedical experimental cell bank	N/A
HEK293T-mito-V5-APEX2 cell line	This paper	N/A
HEK293T-SS-HA-HRP-KDEL cell line	This paper	N/A
HeLa-SS-HA-HRP-KDEL cell line	This paper	N/A
SH-SY5Y-SS-HA-HRP-eGFP-KDEL cell line	This paper	N/A
**Oligonucleotides**		
Primer for pDispaly-SS-HRP-KDEL<br>Fwd: AGGACGAGCTGTAATGAAATG CTGTGGGCCAGGA<br>Rev: CATTACAGCTCGTCCTTCAGA TCCTCTTCTGAGATGAGTTT	This paper	N/A
Primer for pLX304-SS-HRP-KDEL and pLX304-SS-HA-HRP-eGFP-KDEL<br>Fwd: CTTCGAAGGATCCGCCACCAT GGAGACAGACACACTCCTG<br>Rev: GCTAGCTTACAGCTCGTCCTT CAGATCCT	This paper	N/A
**Recombinant DNA**		
pDisplay-SS-HRP-KDEL	This paper	N/A
pLX304-SS-HRP-KDEL	This paper	N/A

pLX304-SS-HA-HRP-eGFP-KDEL	This paper	N/A
**Software and Algorithms**		
MaxQuant software (Version 1.6.2.3)	Cox, J. and Mann, M. 2008	https://www.maxquant.org/
ImageJ (version 1.50e)	Schindelin, J., et al, 2012	https://imagej.nih.gov/ij/
R studio (version 4.1.2)	RStudio Team, 2020	https://www.rstudio.com/

References

[1] Bogenhagen, D. F. & Haley, J. D. Pulse-chase SILAC-based analyses reveal selective oversynthesis and rapid turnover of mitochondrial protein components of respiratory complexes. *J Biol Chem* **295**, 2544-2554 (2020).
