# Supplementary material for: Spatially resolved mapping of proteome turnover dynamics with subcellular precision
Source: Nat Commun. 2023 Nov 8;14:7217. doi: 10.1038/s41467-023-42861-8 (PMC10632371; doi:10.1038/s41467-023-42861-8)
Supplement: Supplementary file 3 — Reporting Summary [file 41467_2023_42861_MOESM3_ESM.pdf]

nature portfolio

Corresponding author(s): Peng Zou

Last updated by author(s): Oct 19, 2023

Reporting Summary

Nature Portfolio wishes to improve the reproducibility of the work that we publish. This form provides structure for consistency and transparency in reporting. For further information on Nature Portfolio policies, see our Editorial Policies and the Editorial Policy Checklist.

Statistics

For all statistical analyses, confirm that the following items are present in the figure legend, table legend, main text, or Methods section.

n/a	Confirmed	
☐	☒	The exact sample size (*n*) for each experimental group/condition, given as a discrete number and unit of measurement
☐	☒	A statement on whether measurements were taken from distinct samples or whether the same sample was measured repeatedly
☐	☒	The statistical test(s) used AND whether they are one- or two-sided<br>*Only common tests should be described solely by name; describe more complex techniques in the Methods section.*
☐	☒	A description of all covariates tested
☐	☒	A description of any assumptions or corrections, such as tests of normality and adjustment for multiple comparisons
☐	☒	A full description of the statistical parameters including central tendency (e.g. means) or other basic estimates (e.g. regression coefficient) AND variation (e.g. standard deviation) or associated estimates of uncertainty (e.g. confidence intervals)
☐	☒	For null hypothesis testing, the test statistic (e.g. *F*, *t*, *r*) with confidence intervals, effect sizes, degrees of freedom and *P* value noted<br>*Give P values as exact values whenever suitable.*
☐	☒	For Bayesian analysis, information on the choice of priors and Markov chain Monte Carlo settings
☐	☒	For hierarchical and complex designs, identification of the appropriate level for tests and full reporting of outcomes
☐	☒	Estimates of effect sizes (e.g. Cohen's *d*, Pearson's *r*), indicating how they were calculated

Our web collection on statistics for biologists contains articles on many of the points above.

Software and code

Policy information about availability of computer code

Data collection	Lab View 2015; Image Lab
Data analysis	Excel 2019; ImageJ 1.8.0; MaxQuant v1.6.10; Graphpad prism 9.0

For manuscripts utilizing custom algorithms or software that are central to the research but not yet described in published literature, software must be made available to editors and reviewers. We strongly encourage code deposition in a community repository (e.g. GitHub). See the Nature Portfolio guidelines for submitting code & software for further information.

Data

Policy information about availability of data

All manuscripts must include a data availability statement. This statement should provide the following information, where applicable:

- Accession codes, unique identifiers, or web links for publicly available datasets
- A description of any restrictions on data availability
- For clinical datasets or third party data, please ensure that the statement adheres to our policy

In the revised manuscript, we have added a "data availability" section under Methods.
The mass spectrometry proteomics data have been deposited to the ProteomeXchange Consortium via the PRIDE partner repository with the dataset identifier PXD037569. The username of the reviewer account for our PRIDE database is “reviewer_pxd037569@ebi.ac.uk”, with password “FRruTOgl”. The raw data of uncropped gel and bar charts are provided in the Source Data file.

Research involving human participants, their data, or biological material

Policy information about studies with human participants or human data. See also policy information about sex, gender (identity/presentation), and sexual orientation and race, ethnicity and racism.

Reporting on sex and gender	not related
Reporting on race, ethnicity, or other socially relevant groupings	not related
Population characteristics	not related
Recruitment	not related
Ethics oversight	not related

Note that full information on the approval of the study protocol must also be provided in the manuscript.

Field-specific reporting

Please select the one below that is the best fit for your research. If you are not sure, read the appropriate sections before making your selection.

[x] Life sciences [ ] Behavioural & social sciences [ ] Ecological, evolutionary & environmental sciences

For a reference copy of the document with all sections, see nature.com/documents/nr-reporting-summary-flat.pdf

Life sciences study design

All studies must disclose on these points even when the disclosure is negative.

Sample size	No sample size calculation was performed. Each MS experiment used of two 10-cm dishes of HEK293T, HeLa or SH-SY5Y cells. Western blot used one cell of 6-wells dish of HEK293T, HeLa or SH-SY5Y cells for each sample. Immunofluorescence used one cell of 24-wells dish of HEK293T, HeLa or SH-SY5Y cells for each sample.
Data exclusions	No data were excluded.
Replication	We performed duplicated and triplicated quantitative MS experiments, which are typical for MS proteomics analysis. Each western blot and imaging fluorescence experiments were also taken for at least 2 replicates. The replicates of each experiment showed good reproducibility, and all attempts at replication were successful.
Randomization	For the experiments taken simultaneously, the cells were from one dish of cells of the previous generation. Thus, the cells for them were randomly distributed to each condition.
Blinding	Mass spec sample preparation and analysis were performed by different experimenters. The MS instrument operator was not informed of the details of proteomic experimental design (control experiments, sample preparation workflow, etc.), thus providing an unbiased analysis of the MS data.

Reporting for specific materials, systems and methods

We require information from authors about some types of materials, experimental systems and methods used in many studies. Here, indicate whether each material, system or method listed is relevant to your study. If you are not sure if a list item applies to your research, read the appropriate section before selecting a response.

Materials & experimental systems

n/a	Involved in the study	
[ ]	[x]	Antibodies
[ ]	[x]	Eukaryotic cell lines
[x]	[ ]	Palaeontology and archaeology
[x]	[ ]	Animals and other organisms
[x]	[ ]	Clinical data
[x]	[ ]	Dual use research of concern
[x]	[ ]	Plants

Methods

n/a	Involved in the study	
[x]	[ ]	ChIP-seq
[x]	[ ]	Flow cytometry
[x]	[ ]	MRI-based neuroimaging

Antibodies

Antibodies used	Mouse anti-TOMM20 monoclonal antibody, Abcam, ab56783<br>Rabbit anti-V5 polyclonal antibody, Abcam, ab9116<br>Rabbit anti-calnexin polyclonal antibody, Abcam, ab22595<br>Rabbit anti-Bip polyclonal antibody, Abcam, ab32618<br>Chicken anti-MAP2 polyclonal antibody, Abcam, ab5392<br>Mouse anti-HA monoclonal antibody, Biodragon, B1003<br>Mouse anti-V5 monoclonal antibody, Biodragon, B1005<br>Mouse anti-GFP monoclonal antibody, Biodragon, B1152<br>Goat anti-Rabbit-Alexa Fluor 488 IgG(H+L), ThermoFisher, A-11034<br>Goat anti-Rabbit-Alexa Fluor 568 IgG(H+L), ThermoFisher, A-11036<br>Goat anti-Mouse-Alexa Fluor 488 IgG(H+L), ThermoFisher, A-11029<br>Goat anti-Mouse-Alexa Fluor 568 IgG(H+L), ThermoFisher, A-11031<br>Goat anti-Chicken-Alexa Fluor 488 IgG(H+L), ThermoFisher, A-11039<br>Streptavidin-Alexa Fluor 647, ThermoFisher, S21374<br>Streptavidin-HRP conjugate, ThermoFisher, 21124<br>HRP-conjugated goat anti mouse IgG, Biodragon, BF03001
Validation	Validation of these antibodies could be found on the websites of the suppliers.

Eukaryotic cell lines

Policy information about cell lines and Sex and Gender in Research

Cell line source(s)	HEK293T cells were from American Type Culture Collection (ATCC) . HeLa cells and SH-SY5Y cells were from Chinese biomedical experimental cell bank. Stable cell lines were generated by lenti virus infection.
Authentication	Stable cell lines expressing APEX2 or HRP were confirmed by fluorescence imaging.
Mycoplasma contamination	Cell lines are PCR-tested positive for mycoplasma contamination.
Commonly misidentified lines<br>(See ICLAC register)	No commonly misidentified cell lines were used in the study.

Plants

Seed stocks	This study does not involve plate samples.
Novel plant genotypes	This study does not involve plate samples.
Authentication	This study does not involve plate samples.
