# Supplementary material for: Spatially resolved mapping of proteome turnover dynamics with subcellular precision
Source: Nat Commun. 2023 Nov 8;14:7217. doi: 10.1038/s41467-023-42861-8 (PMC10632371; doi:10.1038/s41467-023-42861-8)
Supplement: Supplementary file 4 — Description of Additional Supplementary Files [file 41467_2023_42861_MOESM4_ESM.pdf]

File Name: Supplementary Data 1
Description: Mitochondrial prox-SILAC and pulse-SILAC dataset (related to Figure 1).

File Name: Supplementary Data 2
Description: ER lumen prox-SILAC and pulse-SILAC dataset at basal level (related to Figure 2).

File Name: Supplementary Data 3
Description: ER lumen prox-SILAC and pulse-SILAC dataset under ER stress (related to Figure 3).

File Name: Supplementary Data 4
Description: ER lumen prox-SILAC and pulse-SILAC dataset during cell differentiation (related to Figure 4).
