# Supplementary figures and images for: Spatially resolved mapping of proteome turnover dynamics with subcellular precision
Source: Nat Commun. 2023 Nov 8;14:7217. doi: 10.1038/s41467-023-42861-8 (PMC10632371; doi:10.1038/s41467-023-42861-8)

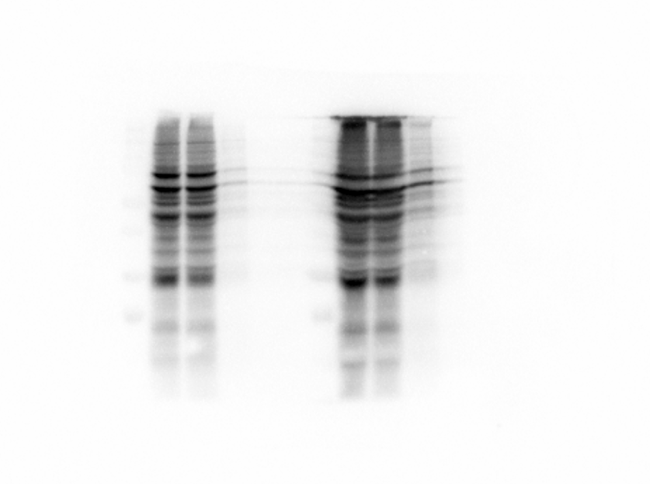

Supplement: Supplementary file 9 — Source Data [file 41467_2023_42861_MOESM9_ESM.zip › uncropped gel image/Supplementary Figure 1/Supplementary Figure 1A-1.tif]

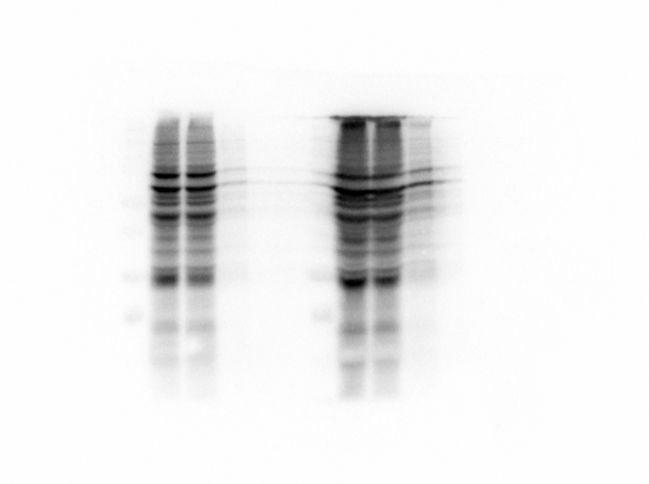

Supplement: Supplementary file 9 — Source Data [file 41467_2023_42861_MOESM9_ESM.zip › uncropped gel image/Supplementary Figure 1/Supplementary Figure 1A-2.tif]

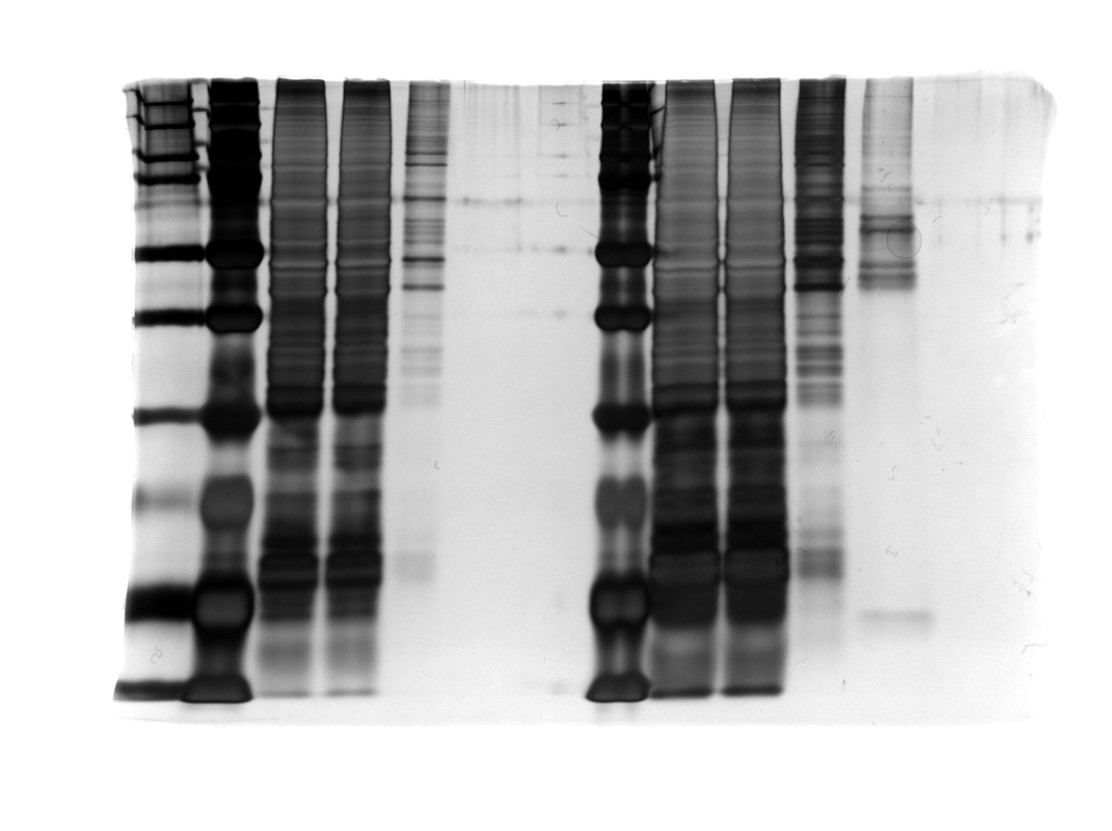

Supplement: Supplementary file 9 — Source Data [file 41467_2023_42861_MOESM9_ESM.zip › uncropped gel image/Supplementary Figure 1/Supplementary Figure 1A-3.jpg]

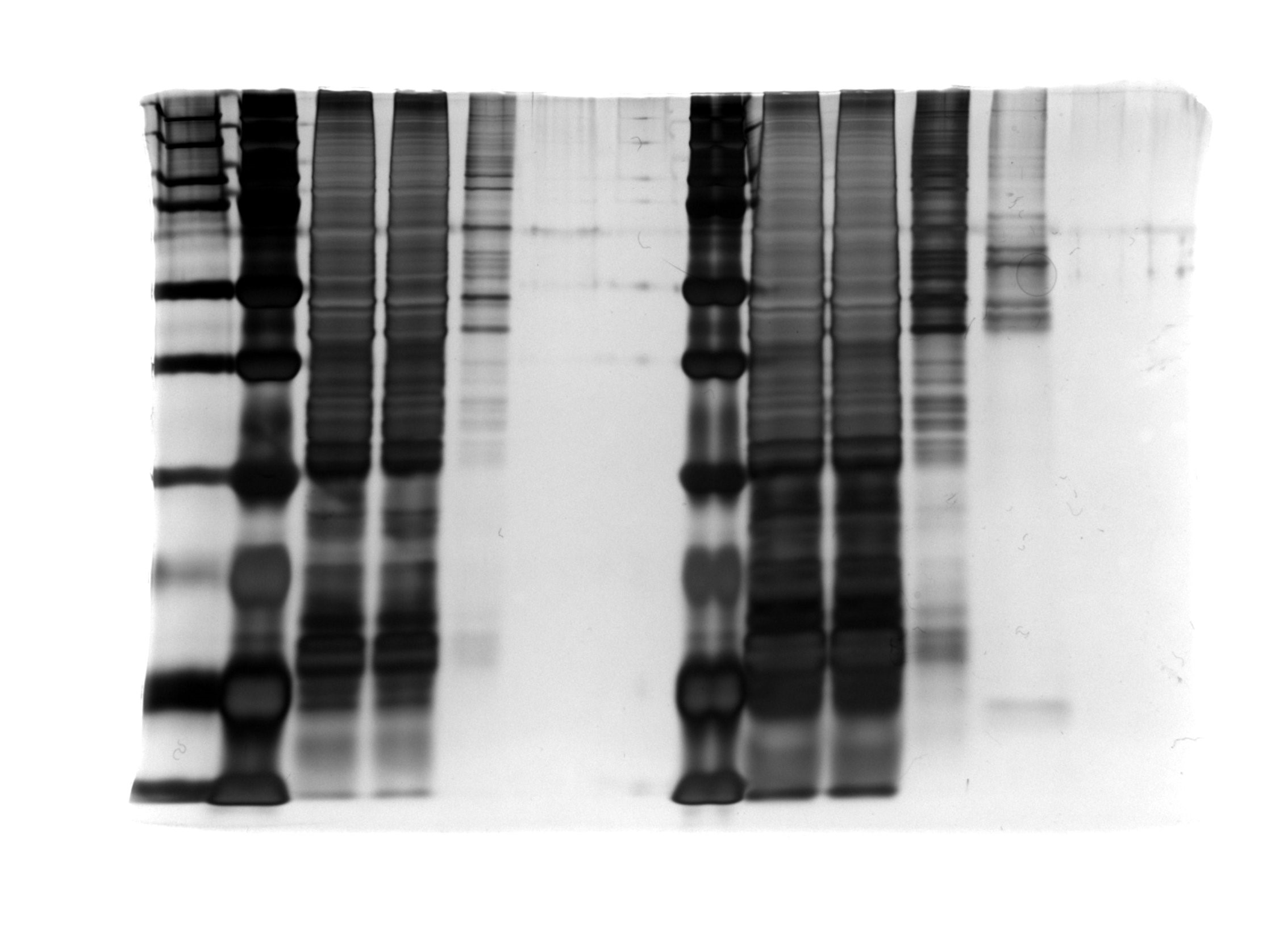

Supplement: Supplementary file 9 — Source Data [file 41467_2023_42861_MOESM9_ESM.zip › uncropped gel image/Supplementary Figure 1/Supplementary Figure 1A-4.jpg]

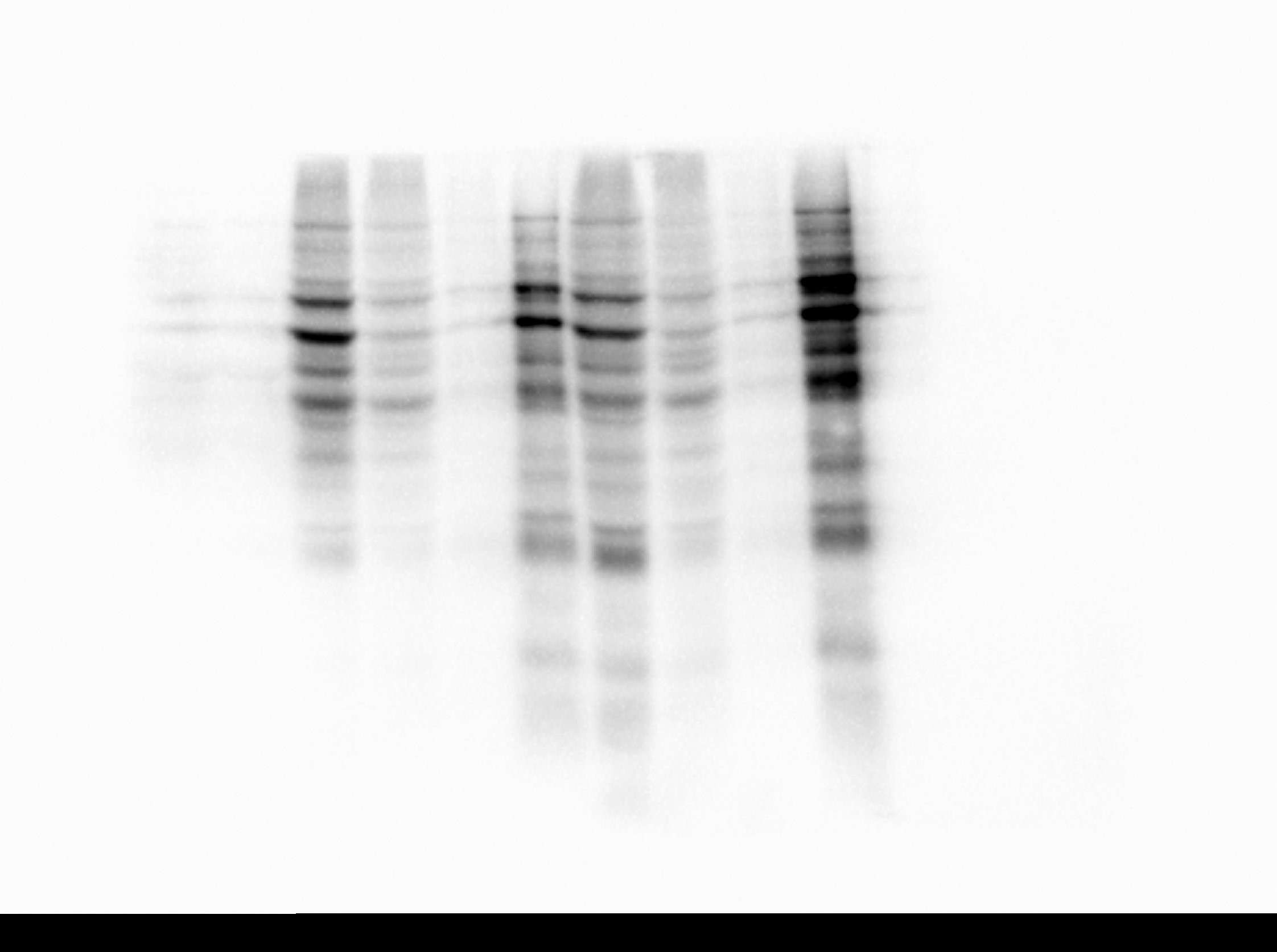

Supplement: Supplementary file 9 — Source Data [file 41467_2023_42861_MOESM9_ESM.zip › uncropped gel image/Supplementary Figure 1/Supplementary Figure 1B-1.jpg]

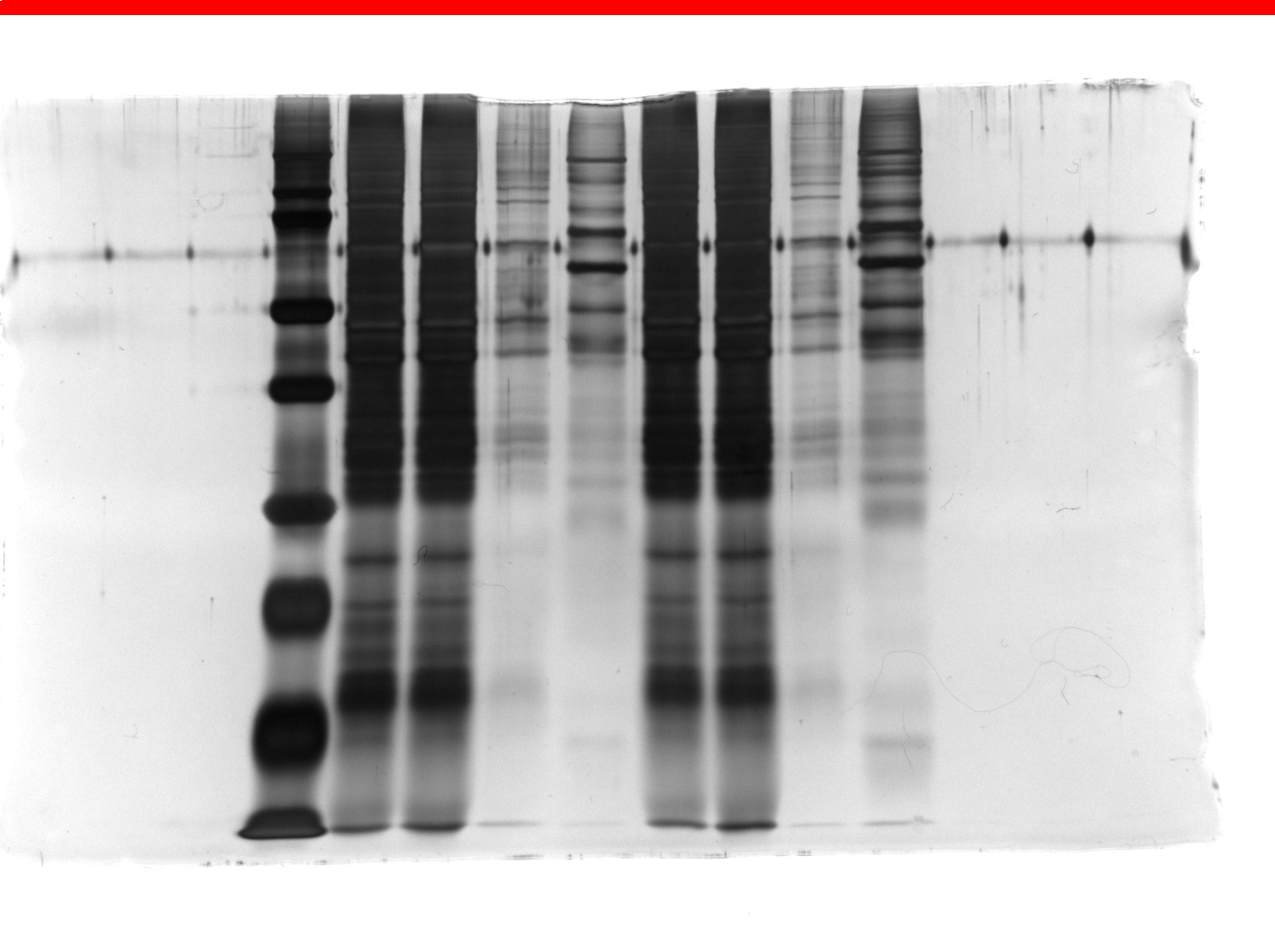

Supplement: Supplementary file 9 — Source Data [file 41467_2023_42861_MOESM9_ESM.zip › uncropped gel image/Supplementary Figure 1/Supplementary Figure 1B-2.jpg]

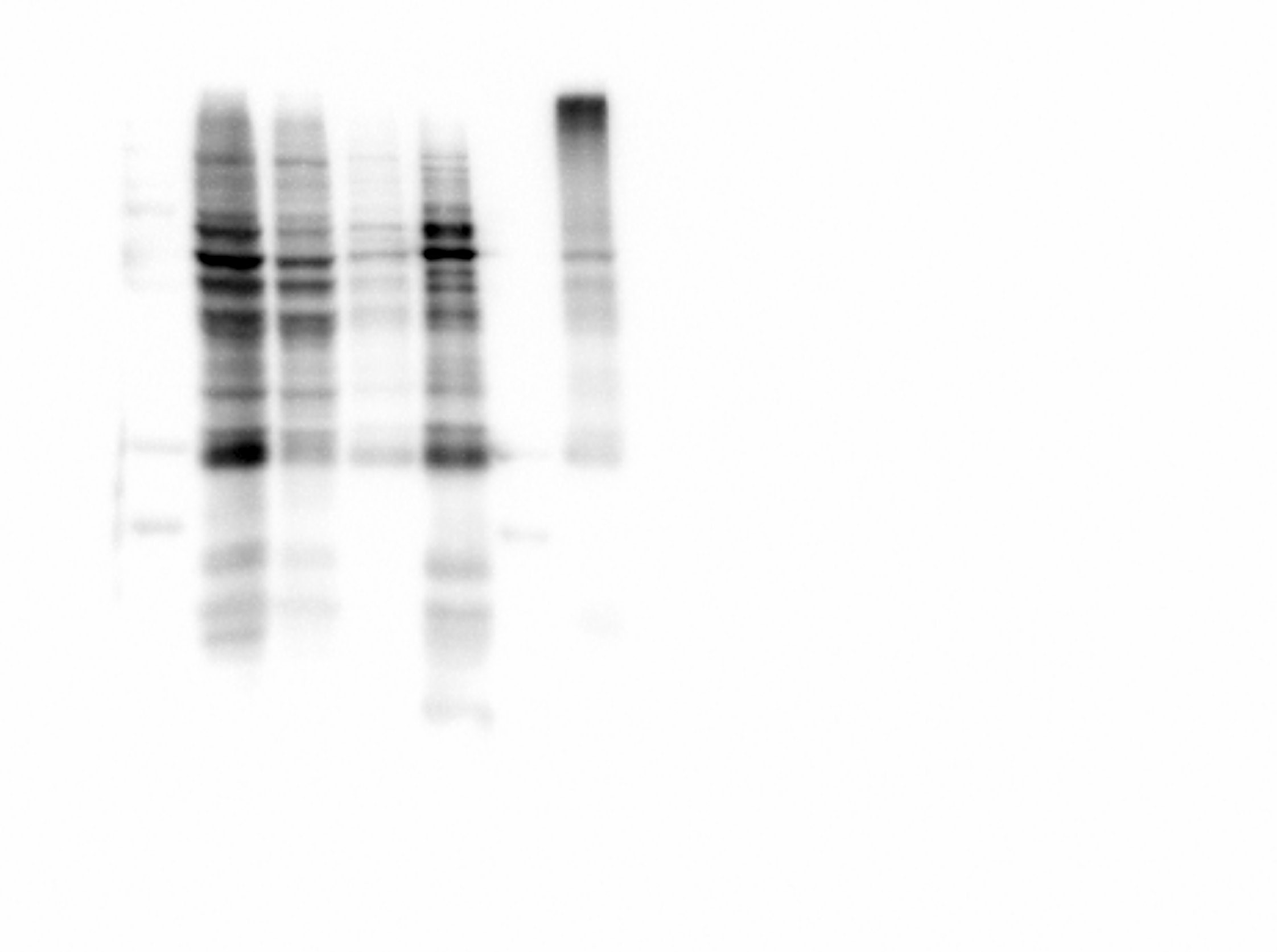

Supplement: Supplementary file 9 — Source Data [file 41467_2023_42861_MOESM9_ESM.zip › uncropped gel image/Supplementary Figure 1/Supplementary Figure 1C-1.jpg]

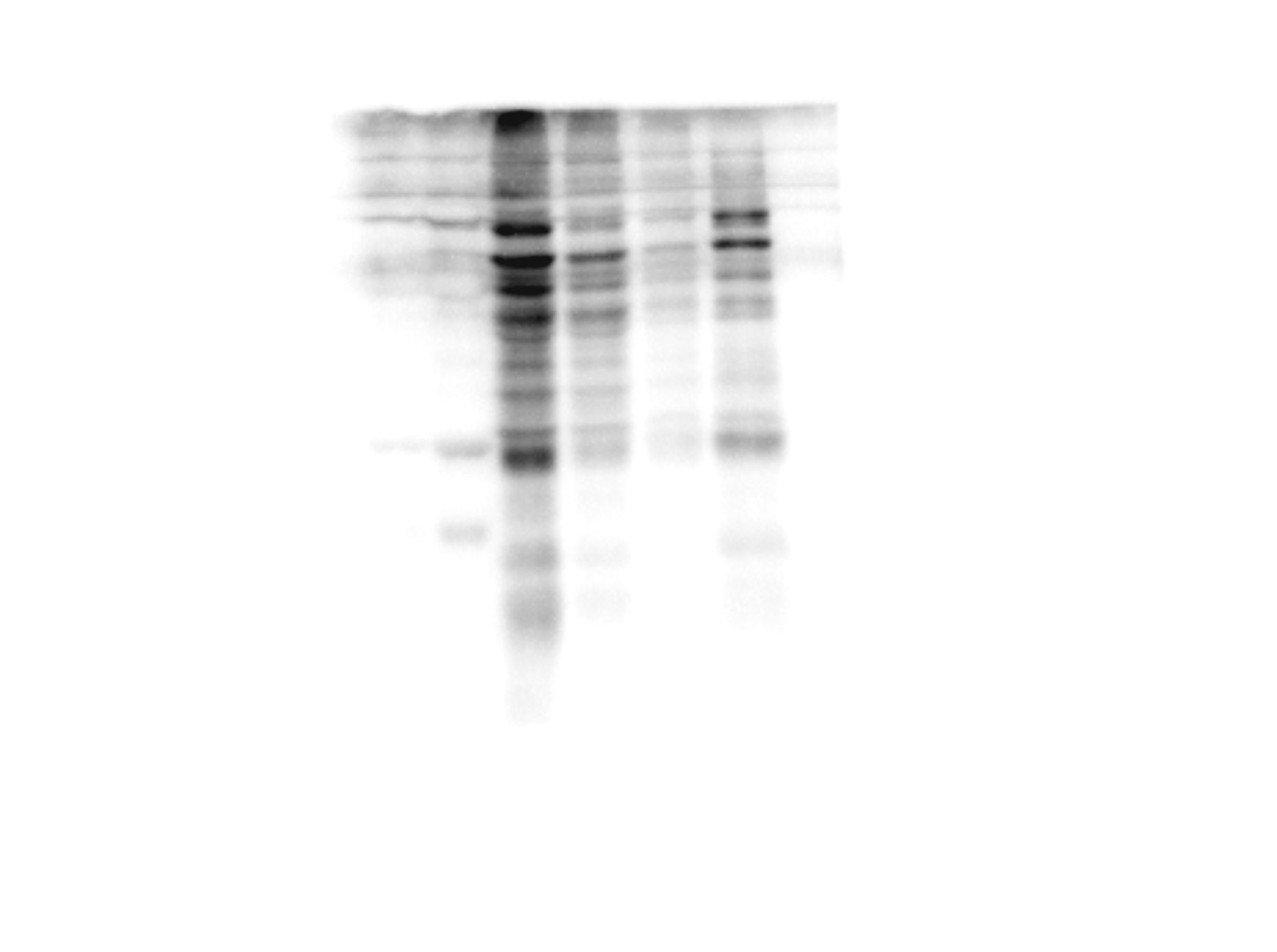

Supplement: Supplementary file 9 — Source Data [file 41467_2023_42861_MOESM9_ESM.zip › uncropped gel image/Supplementary Figure 1/Supplementary Figure 1C-2.jpg]

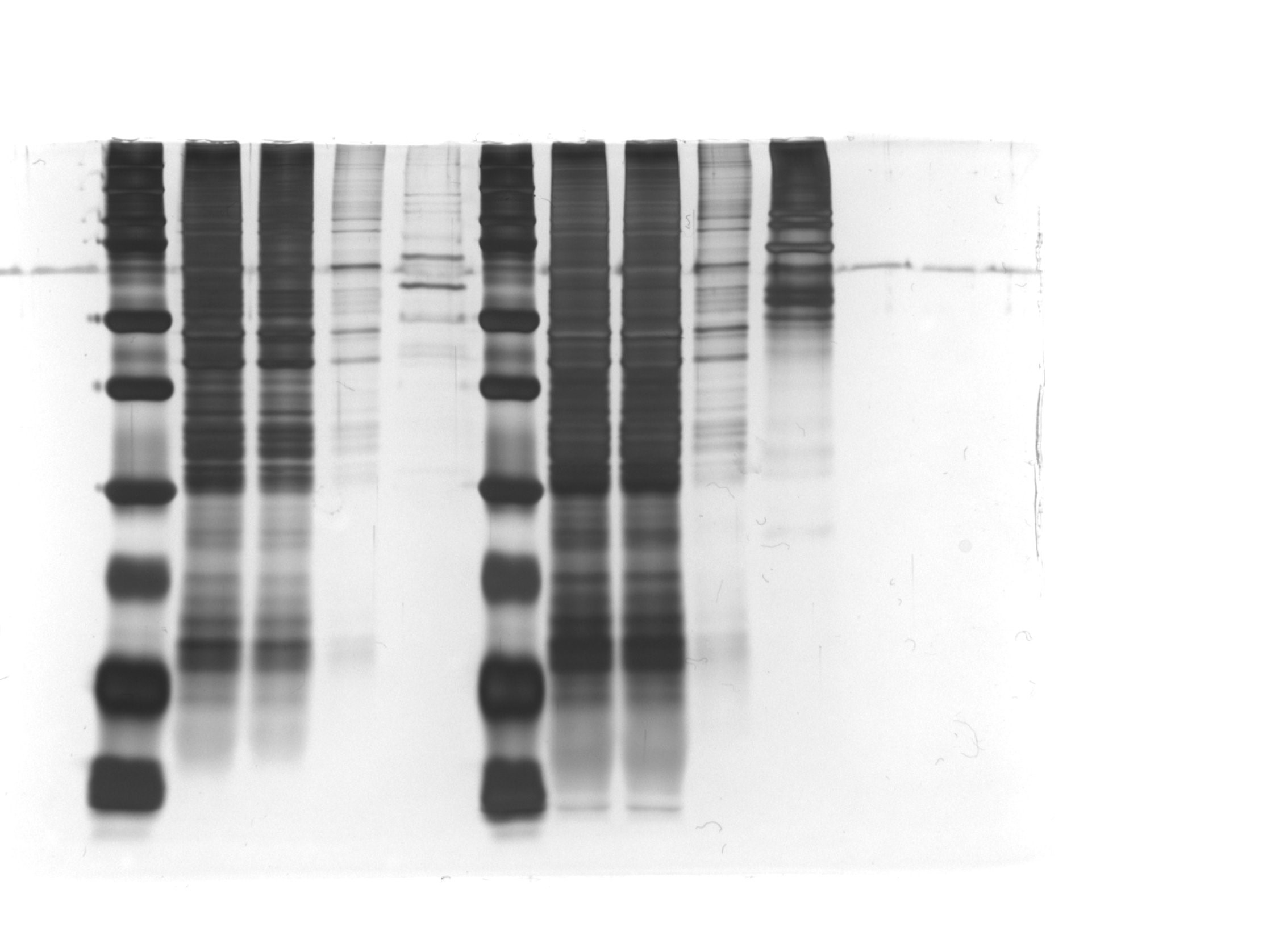

Supplement: Supplementary file 9 — Source Data [file 41467_2023_42861_MOESM9_ESM.zip › uncropped gel image/Supplementary Figure 1/Supplementary Figure 1C-3.jpg]

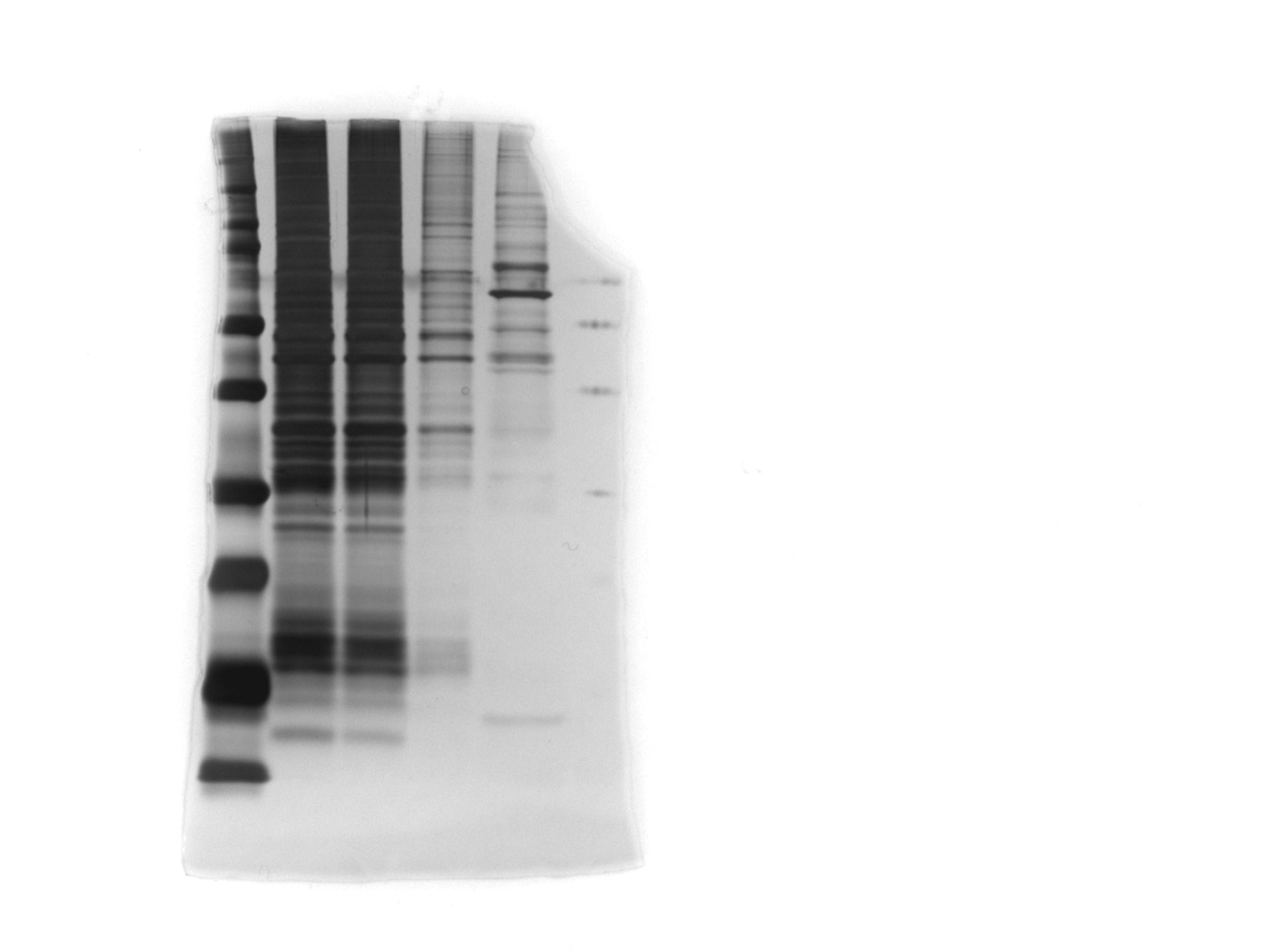

Supplement: Supplementary file 9 — Source Data [file 41467_2023_42861_MOESM9_ESM.zip › uncropped gel image/Supplementary Figure 1/Supplementary Figure 1C-4.jpg]

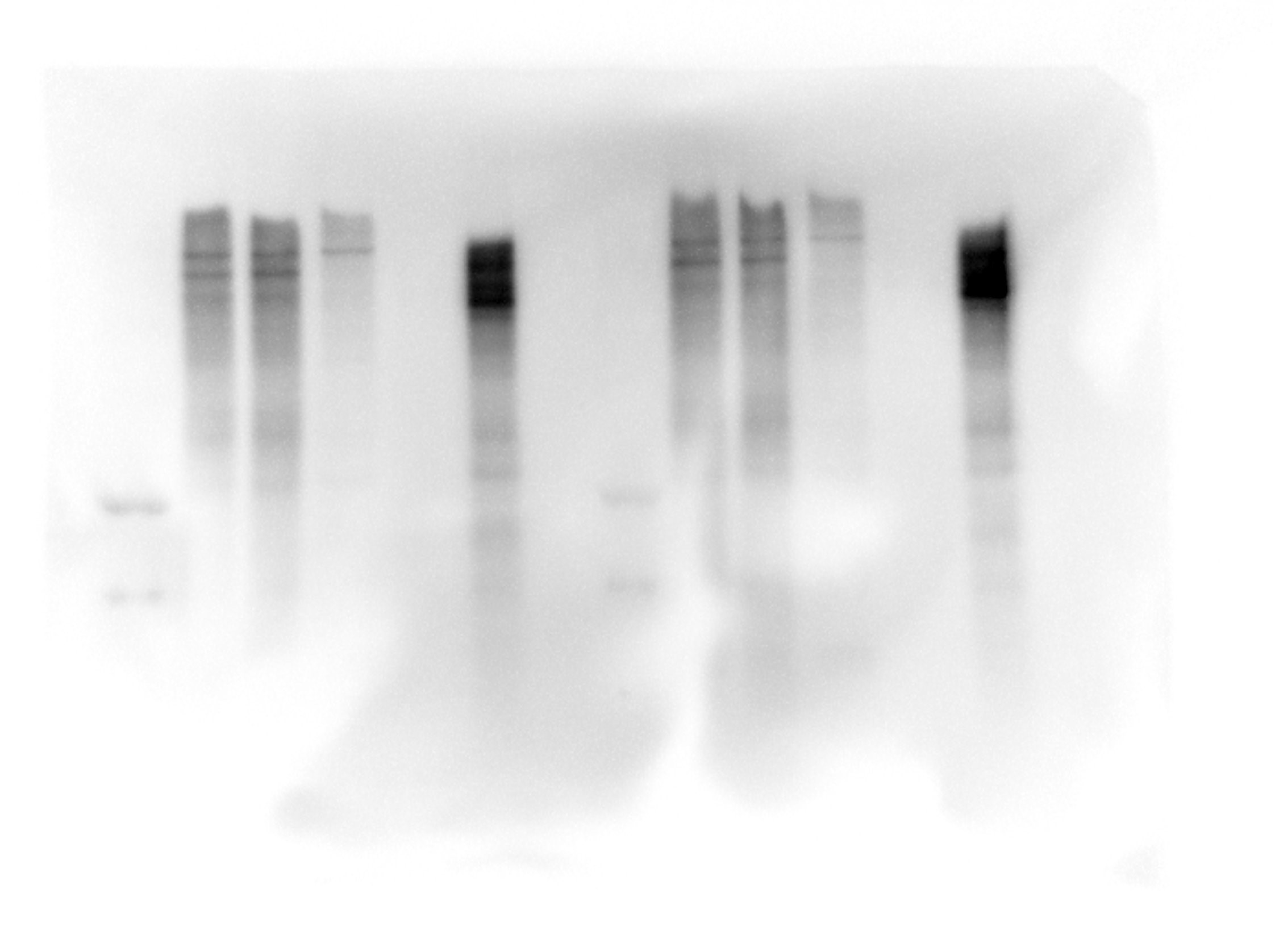

Supplement: Supplementary file 9 — Source Data [file 41467_2023_42861_MOESM9_ESM.zip › uncropped gel image/Supplementary Figure 12/Supplementary Figure 12A-1.jpg]

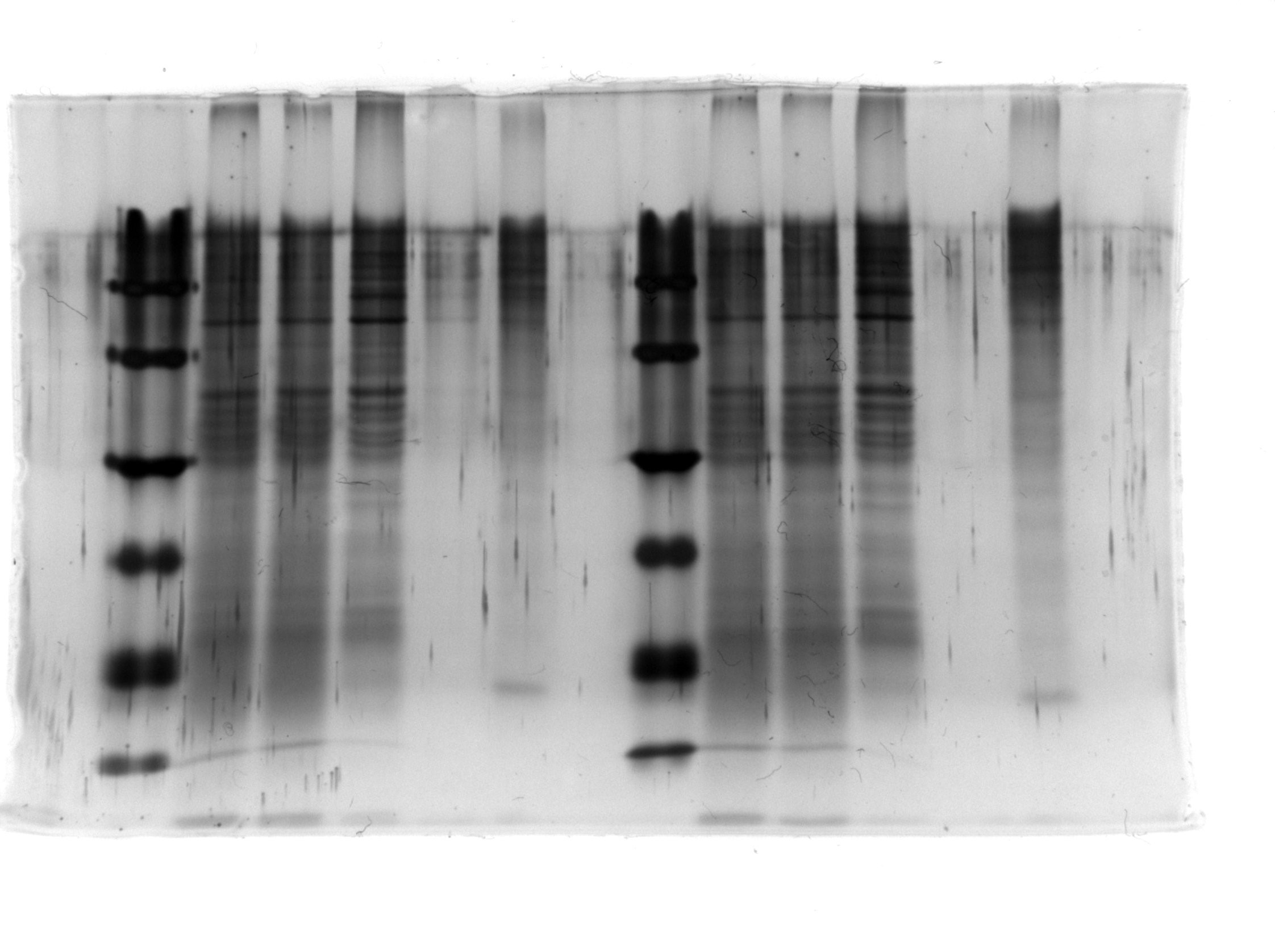

Supplement: Supplementary file 9 — Source Data [file 41467_2023_42861_MOESM9_ESM.zip › uncropped gel image/Supplementary Figure 12/Supplementary Figure 12A-3.jpg]

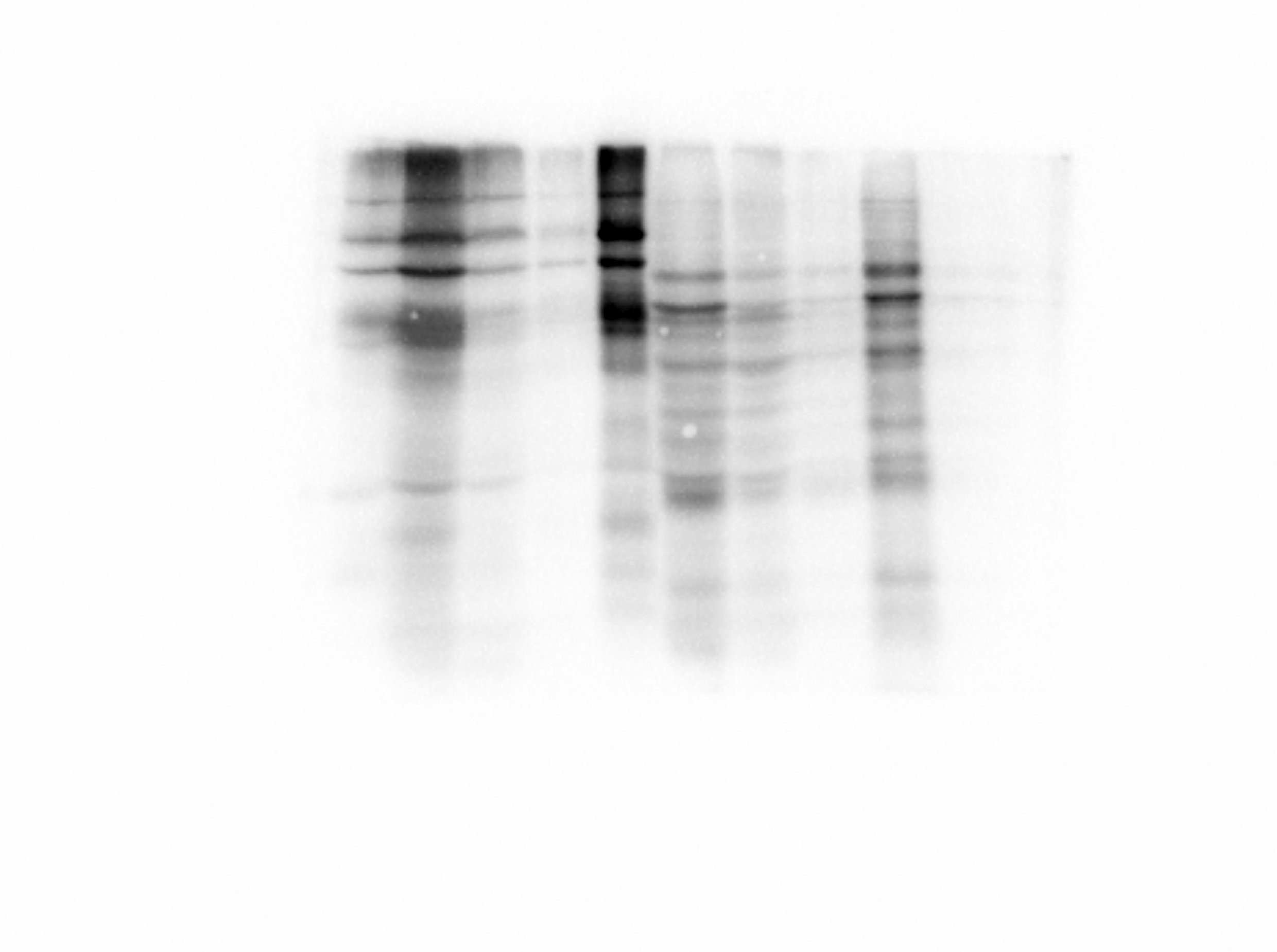

Supplement: Supplementary file 9 — Source Data [file 41467_2023_42861_MOESM9_ESM.zip › uncropped gel image/Supplementary Figure 12/Supplementary Figure 12B-1.jpg]

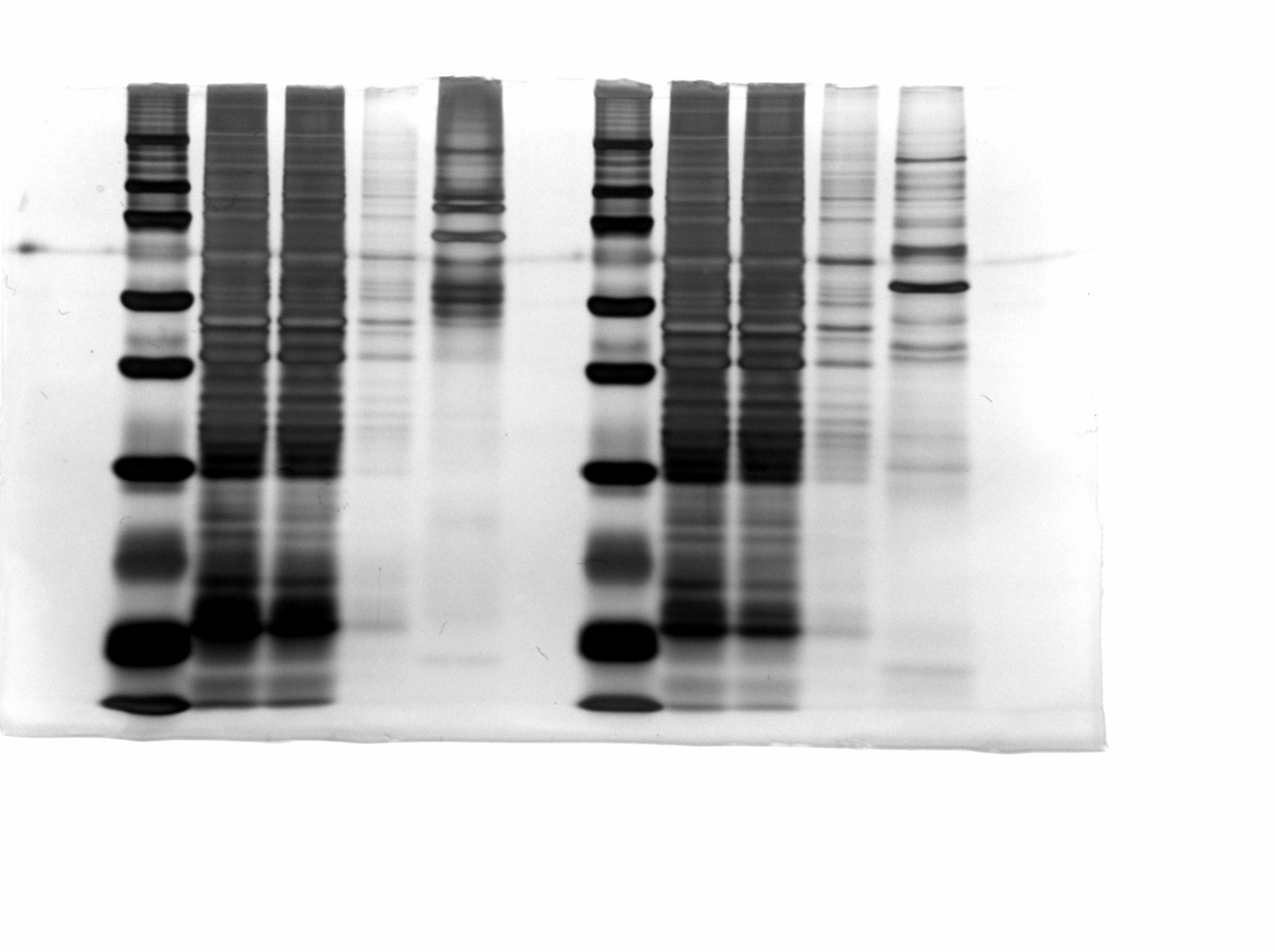

Supplement: Supplementary file 9 — Source Data [file 41467_2023_42861_MOESM9_ESM.zip › uncropped gel image/Supplementary Figure 12/Supplementary Figure 12B-3.jpg]

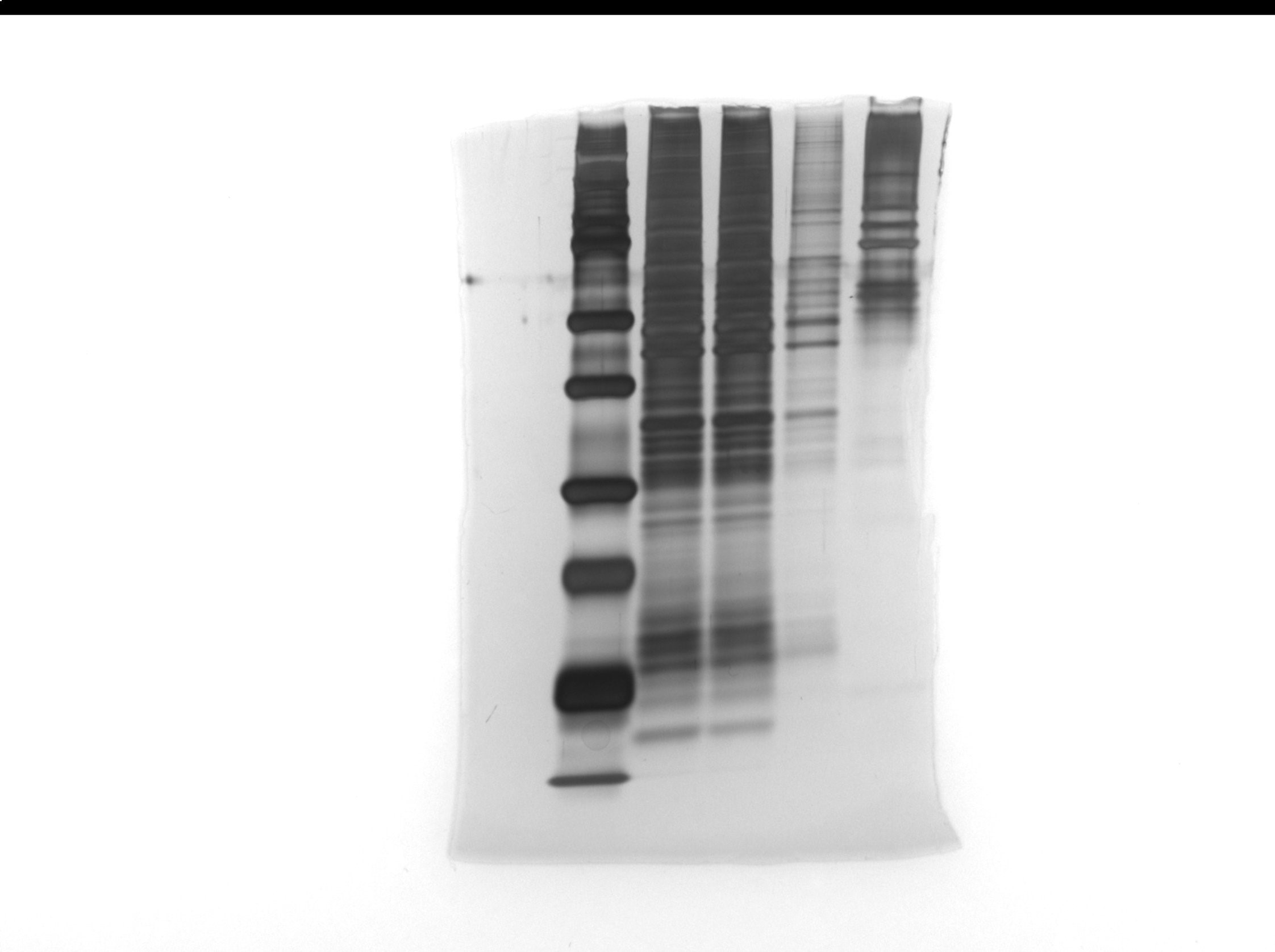

Supplement: Supplementary file 9 — Source Data [file 41467_2023_42861_MOESM9_ESM.zip › uncropped gel image/Supplementary Figure 12/Supplementary Figure 12B-4.jpg]

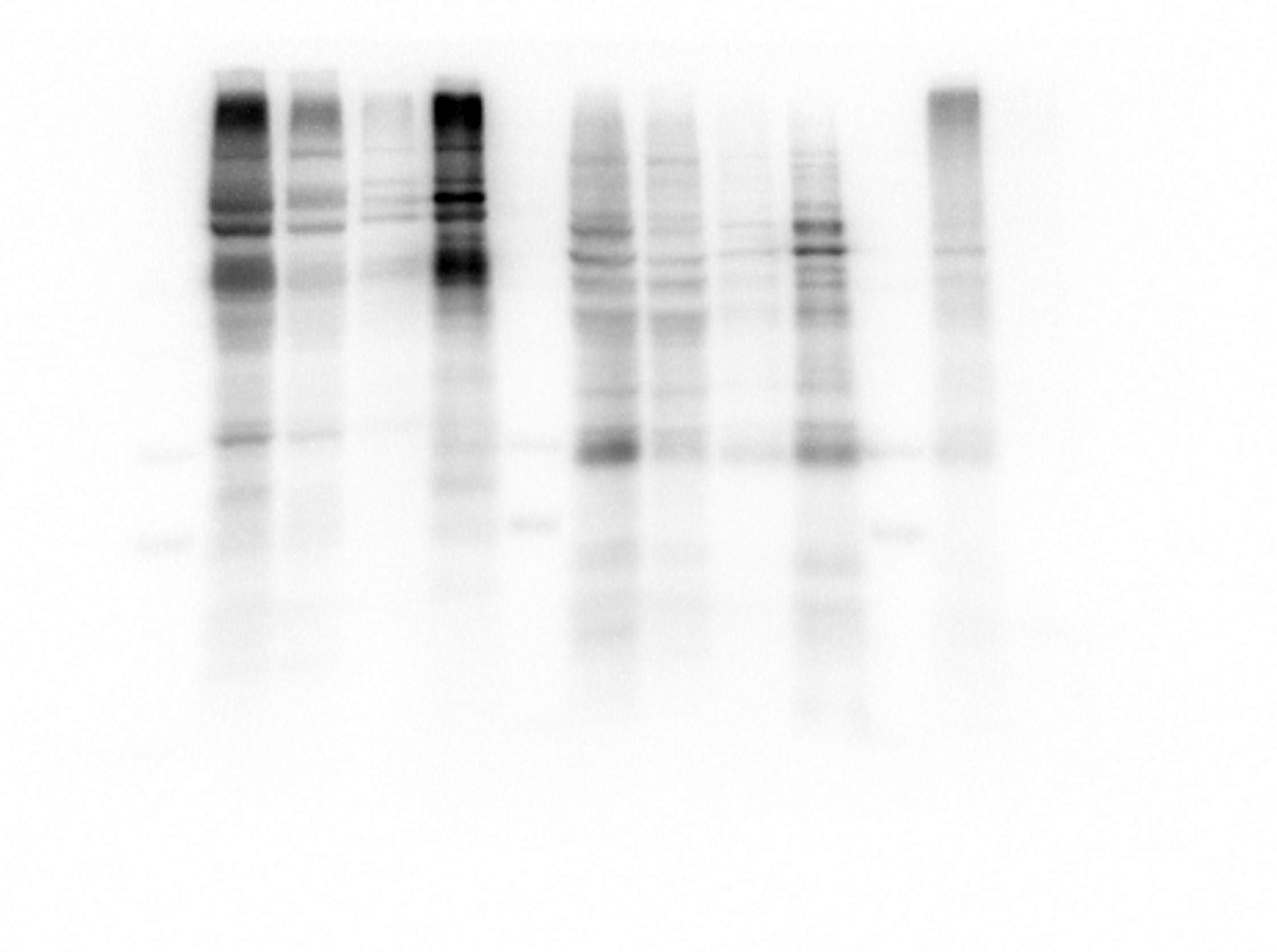

Supplement: Supplementary file 9 — Source Data [file 41467_2023_42861_MOESM9_ESM.zip › uncropped gel image/Supplementary Figure 12/Supplementary Figure 12C-1.jpg]

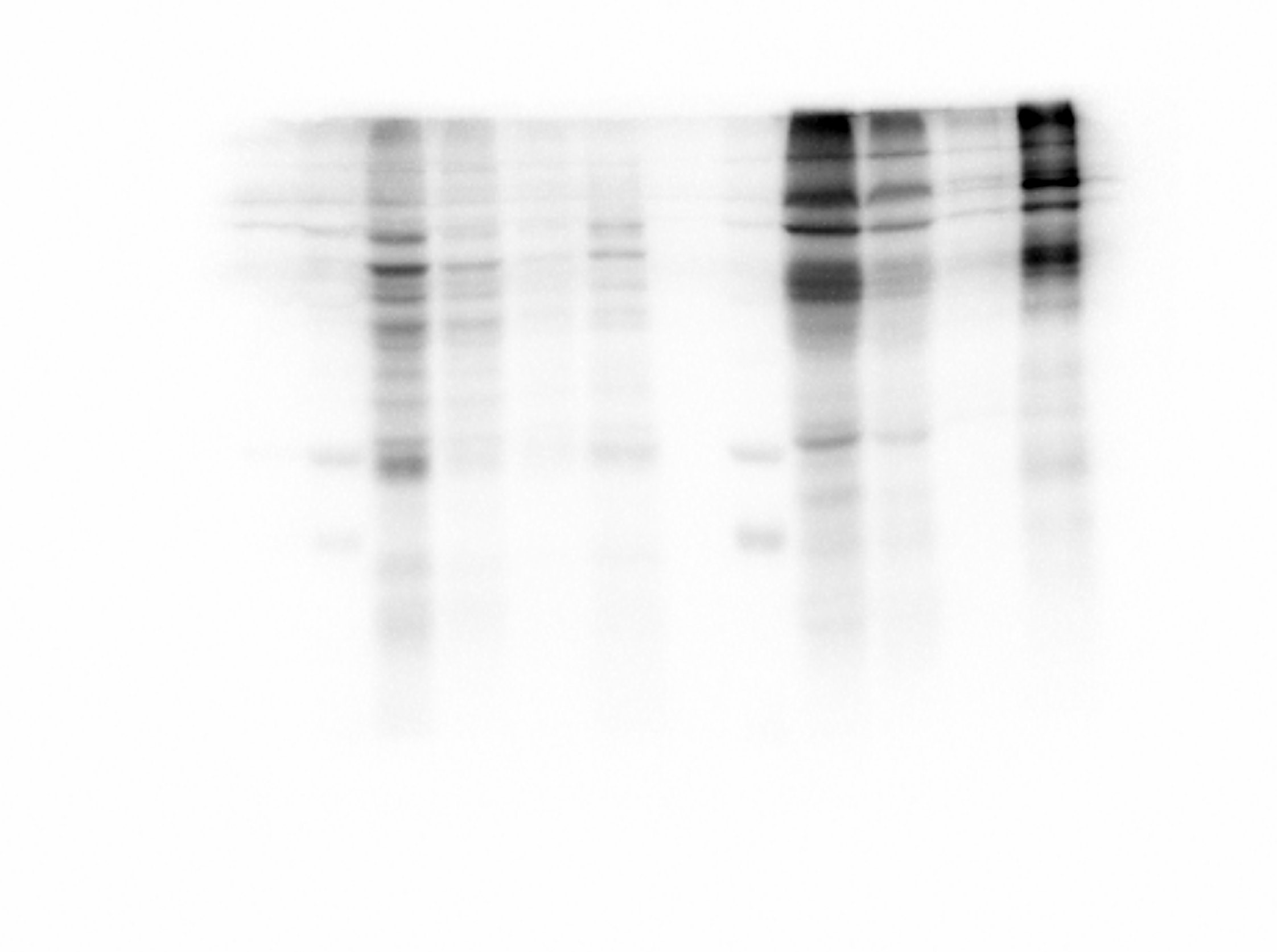

Supplement: Supplementary file 9 — Source Data [file 41467_2023_42861_MOESM9_ESM.zip › uncropped gel image/Supplementary Figure 12/Supplementary Figure 12C-2.jpg]

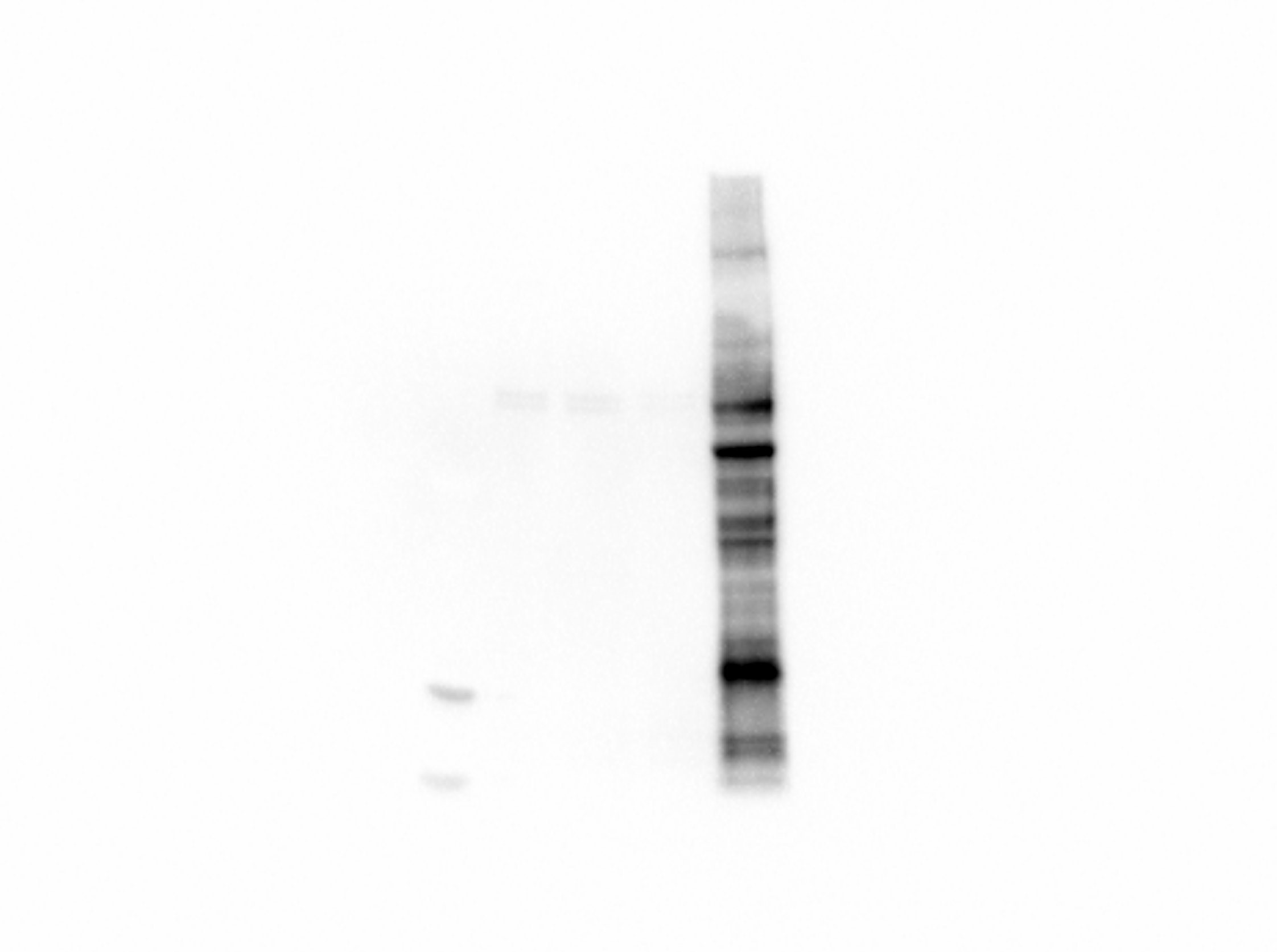

Supplement: Supplementary file 9 — Source Data [file 41467_2023_42861_MOESM9_ESM.zip › uncropped gel image/Supplementary Figure 2/Supplementary Figure 2-1.jpg]

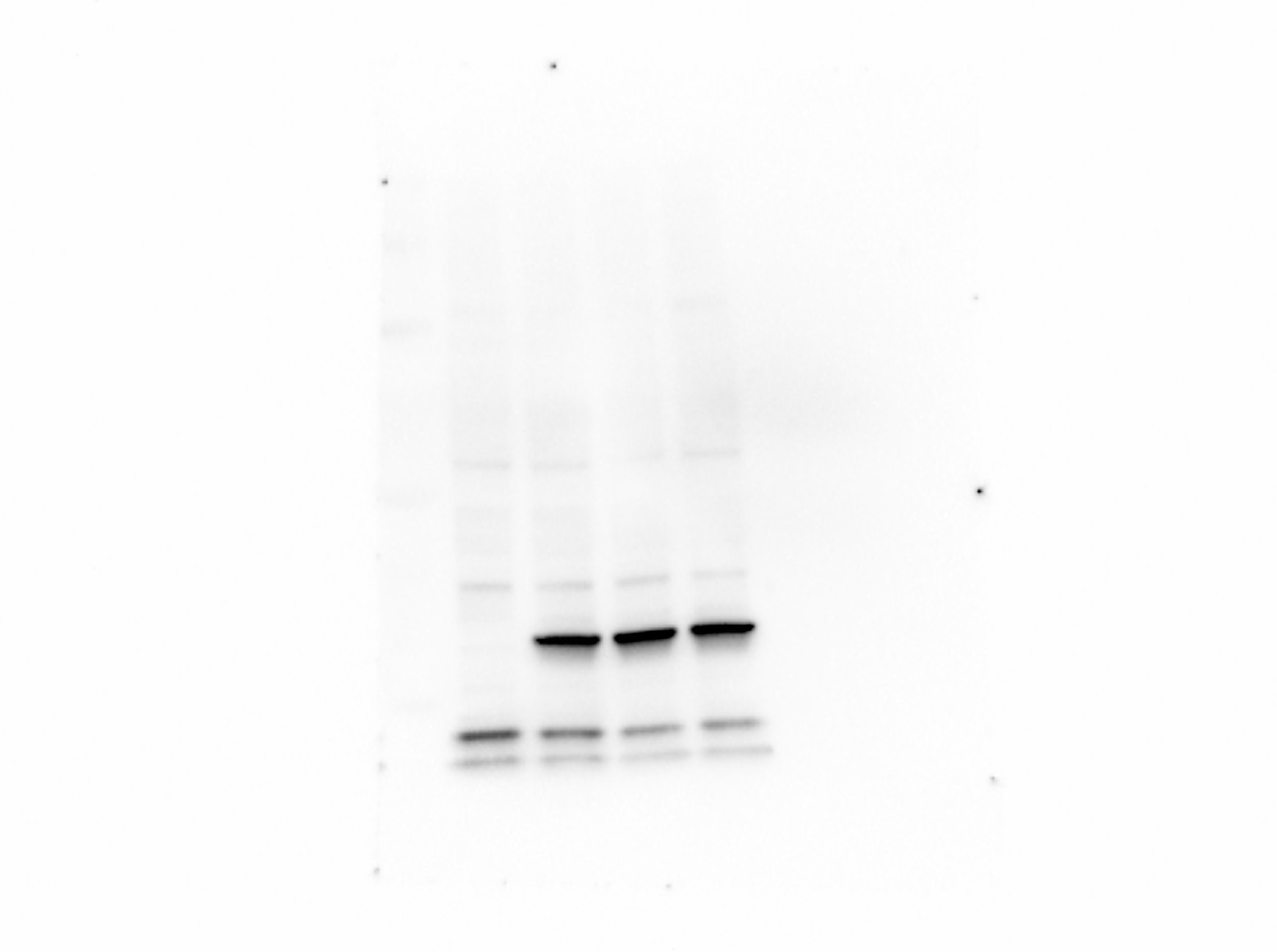

Supplement: Supplementary file 9 — Source Data [file 41467_2023_42861_MOESM9_ESM.zip › uncropped gel image/Supplementary Figure 2/Supplementary Figure 2-2.jpg]

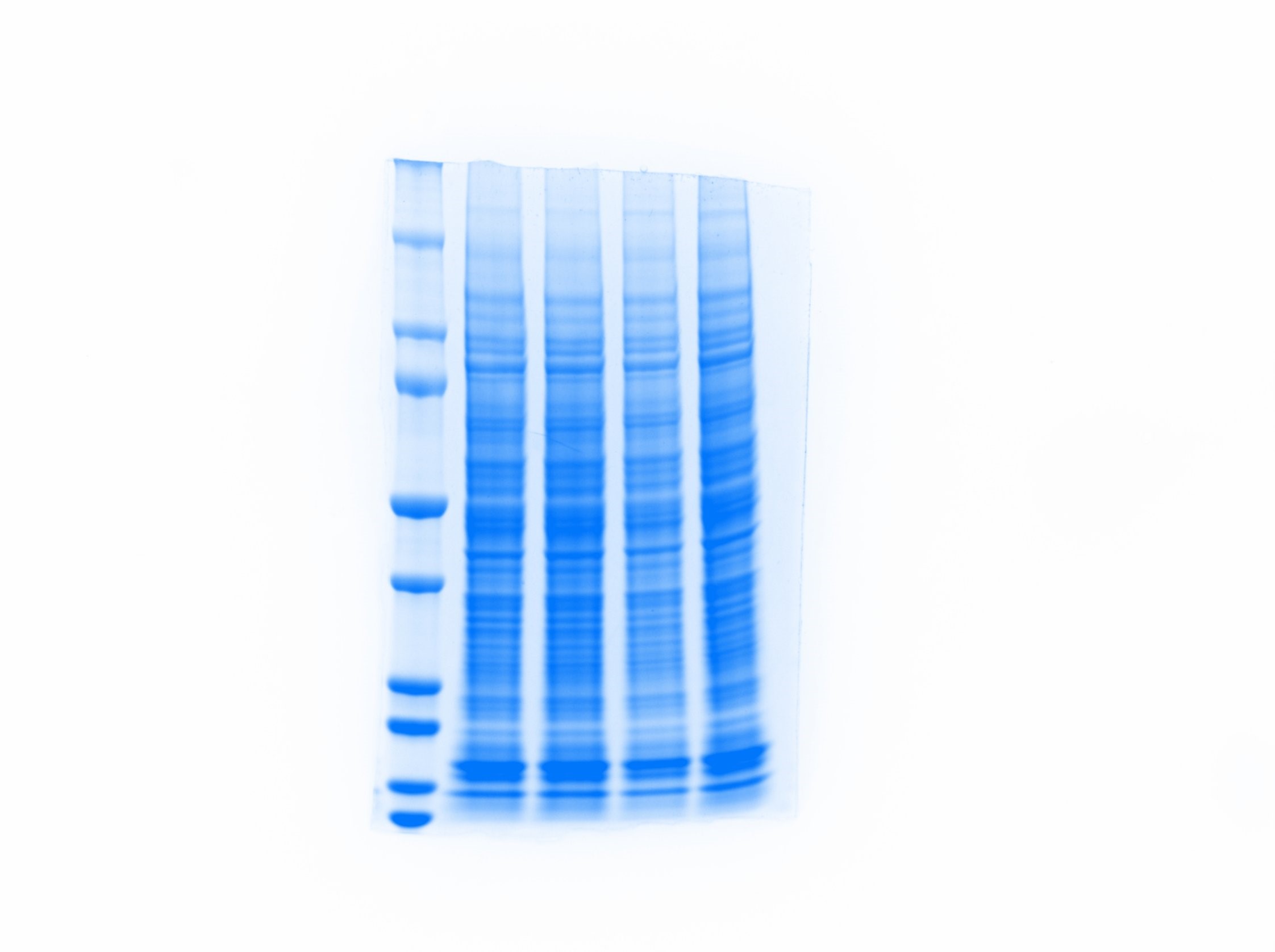

Supplement: Supplementary file 9 — Source Data [file 41467_2023_42861_MOESM9_ESM.zip › uncropped gel image/Supplementary Figure 2/Supplementary Figure 2-3.jpg]

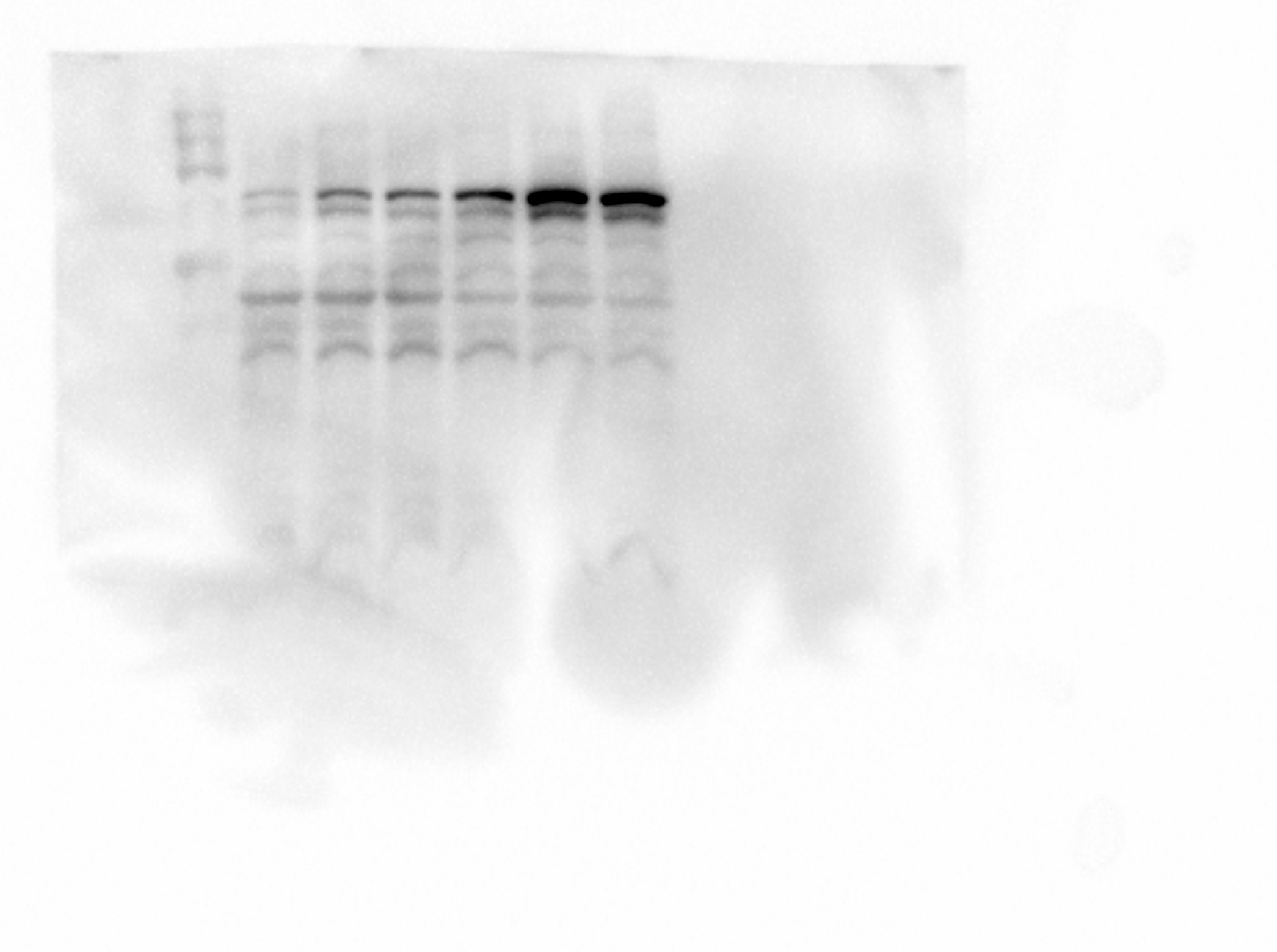

Supplement: Supplementary file 9 — Source Data [file 41467_2023_42861_MOESM9_ESM.zip › uncropped gel image/Supplementary Figure 21/Supplementary Figure 21-1.jpg]

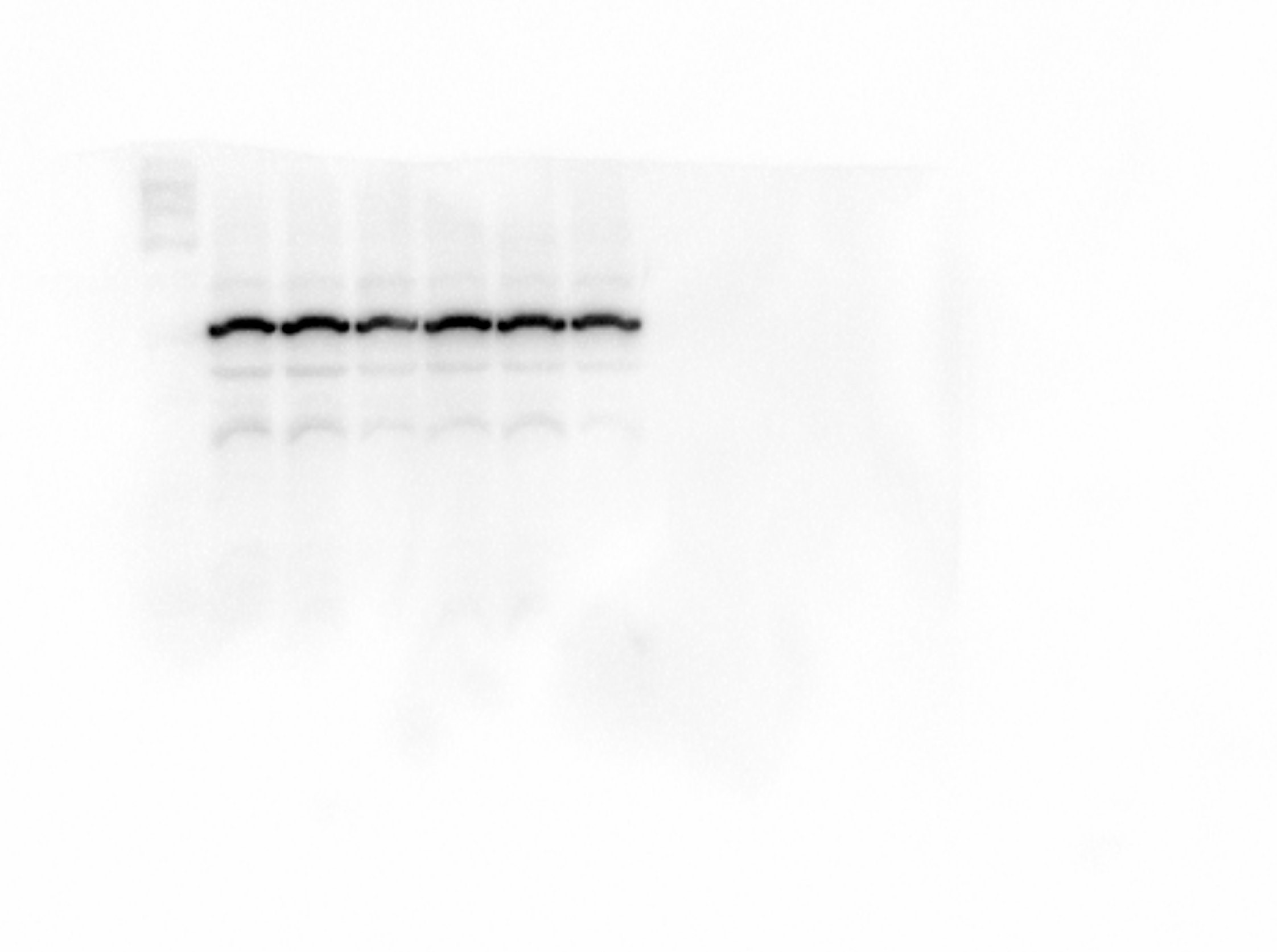

Supplement: Supplementary file 9 — Source Data [file 41467_2023_42861_MOESM9_ESM.zip › uncropped gel image/Supplementary Figure 21/Supplementary Figure 21-2.jpg]
